# PER1 interaction with GPX1 regulates metabolic homeostasis under oxidative stress

**DOI:** 10.1016/j.redox.2020.101694

**Published:** 2020-08-26

**Authors:** Qi Sun, Yunxia Yang, Zhongqiu Wang, Xiao Yang, Yan Gao, Yang Zhao, Wenhao Ge, Junhao Liu, Xi Xu, Wei Guan, Dan Weng, Shiming Wang, Junsong Wang, Jianfa Zhang

**Affiliations:** a Center for Molecular Metabolism, Nanjing University of Science & Technology, Nanjing, 210094, China; b Key Laboratory of Cardiovascular and Cerebrovascular Diseases, Bengbu Medical College, Bengbu, 233030, China; c Affiliated Hospital of Nanjing University of Chinese Medicine, Nanjing, 210029, China; d The Second Hospital of Nanjing, Nanjing Medical University, Nanjing, 210003, China

**Keywords:** PER1, GPX1, ROS, Oxidation stress, Metabolic rhythm

## Abstract

Metabolism serves mammalian feeding and active behavior, and is controlled by circadian clock. The molecular mechanism by which clock factors regulate metabolic homeostasis under oxidative stress is unclear. Here, we have characterized that the daily oxygen consumption rhythm was deregulated in *Per1* deficient mice. *Per1* deficiency impaired daily mitochondrial dynamics and deregulated cellular GPx-related ROS fluctuations in the peripheral organs. We identified that PER1 enhanced GPx activity through PER1/GPX1 interaction in cytoplasm, consequently improving the oxidative phosphorylation efficiency of mitochondria. *Per1* expression was specifically elevated in the fasting peripheral organs for protecting mitochondrial from oxidation stress. These observations reveal that *Per1*-driven mitochondrial dynamics is a critical effector mechanism for the regulation of mitochondrial function in response to oxidation stress.

## Highlights

• PER1 regulates daily metabolic rhythm uncoupled from feeding oscillations.
• *Per1* deficiency impairs mitochondrial dynamics and deregulates ROS fluctuations.
• PER1 interactions with GPX1 and increases mitochondrial ROS clearance.
• Fasting elevates *Per1* expression to protect mitochondrial from oxidation stress.

## Graphical abstract

**Figure:**
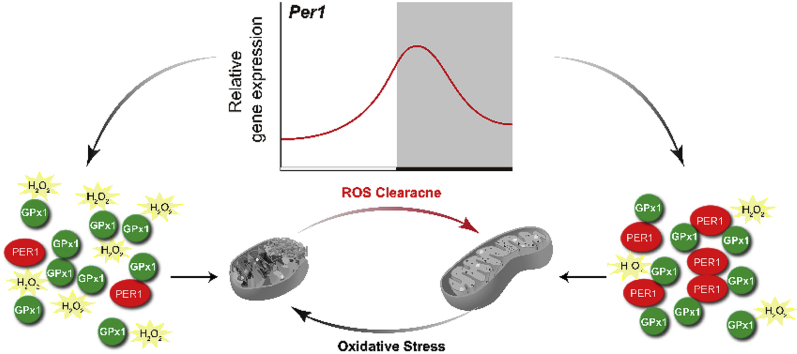

## 1 Introduction

Major behavioral oscillations including active/inactive and feeding/fasting rhythms persist in a daily basis in mammals. Cells/organs maintain adequate metabolic flexibility to facilitate efficient food availability and storage of nutrients [1]. Energy metabolic rate fluctuates over the course of the day, concomitant with daily sleep-wake and fasting-feeding cycles. Energy expenditure and oxygen consumption are elevated in the laboratory rodent during the active period, associating with behaviors such as increasing physical activity and food intake [2,3]. Metabolism serves the mammalian feeding and active behavior. Various observations suggest that 24 h oscillations in specific metabolic parameters are not driven solely by behavioral rhythms, but are instead mediated by endogenous circadian control mechanisms [3,4]. Several metabolic genes exhibit rhythmic expression patterns and are controlled by circadian clock system [[5], [6], [7], [8]]. Metabolism regulation by circadian clocks likely allows anticipation of daily fluctuations in energy demand and/or nutrient availability [[9], [10], [11], [12]].

Mitochondria are the major metabolic nodes providing 90% of cellular energy by converting lipids and carbohydrates into ATP through oxidative phosphorylation [13]. Mitochondria are therefore highly dynamic, and their activity varies according to the cell's nutritional status at different times of the day [14,15]. This flexibility is supposed to be achieved through some possible crosstalk mechanisms between mitochondrial functions and circadian clocks [[16], [17], [18], [19], [20]]. Mitochondria also generate much of the cellular reactive oxygen species (ROS) as a by-product within most mammalian cells [21,22]. Oxidative stress resulting from an excessive ROS production or ineffective antioxidant response promotes mitochondrial dysfunction and affects cell viability by damaging nucleic acids, proteins and lipids [23]. Not surprisingly, oxidative stress has been implicated in multiple pathologies such as cancer, type II diabetes, arteriosclerosis, chronic inflammatory processes, ischemia/reperfusion injury, as well as various neurodegenerative diseases in humans [24].

Despite the exact biological significance of metabolism regulation by circadian clocks, the several fundamental questions remain unanswered regarding the circadian clock factors towards metabolic homeostasis under oxidative stress. In this study, we have uncovered a mechanism by which circadian rhythm PER1 directly regulates GPX1 activity and ROS levels in liver and small intestine, thereby contributing to the daily rhythm of mitochondrial morphology and function. We demonstrated that *Per1* expression was specifically elevated in the fasting peripheral organs for protecting mitochondrial from oxidation stress. Our observations reveal that PER1-driven mitochondrial dynamics are a critical effector mechanism for the regulation of energy substrate utilization in response to daily behavior rhythm and oxidation stress.

## 2 Materials and methods

### 2.1 Cell culture

NIH-3T3 cell lines were cultured in RPMI 1640 medium (Gibco, Darmstadt, Germany) supplemented by 10% fetal bovine serum (FBS) (Gibco, Darmstadt, Germany), 100 μg/ml penicillin and 100 IU/ml streptomycin under humidified and 5% CO_2_ conditions. The cells were transfected in Opti-MEM using Lipofectamine 2000 (ThermoFisher) following manufacturer's protocol. The cells were imaged or fixed 24–36 h post transfection and the genes expressions were determined at 48 h after transfection.

### 2.2 Animal studies

*Per1*^*−/−*^ mice were obtained from Dr. CC Lee at Baylor College of Medicine, Houston, TX, USA [25]. *Per1*^*−/−*^ mice on the 129SvEv background were bred onto the C57BL/6J (Jackson Laboratory, Bar Harbor, ME, USA) background for eight to ten generations (N8 to N10) according to standard genetic protocols. Wild-type (WT, C57BL/6J), *Per1*^*−/−*^ mice were housed in a standard animal maintenance facility under a 12-h/12-h light/dark cycle (lights on at 7 a.m., ZT0; lights off at 7 p.m., ZT12) and provided with food and water ad libitum. For circadian/diurnal studies, mice between 6 and 8 weeks old were sacrificed every 4 h for 24 h. All animal care and use procedures were in accordance with the guidelines of the Institutional Animal Care and Use Committee at Nanjing University of Science and Technology (ACUC-NUST-20160016).

### 2.3 Metabolic studies

Phenomaster Metabolic Cages (PMC) were used to simultaneously quantify energy expenditure, energy intake, locomotor activity and respiratory exchange ratio (RER). For PMC studies, male WT and *Per1*^−/−^ mice fed a ND at 8–10 weeks old were acclimated to the chambers for 4 days and recordings were performed for 2 days, yielding one full 24-h period of data. Feeding and activity data were collected continuously; O_2_ and CO_2_ levels for energy expenditure were collected at 45-min intervals.

### 2.4 Enzyme activity assays

Liver and small intestine tissues were homogenized at 1/9 (w/v) dilution in cold saline at ice-bath. Suspensions were centrifuged at 800×*g* for 10 min at 4 °C to remove nuclei and cell debris. The pellets were discarded, and supernatants were used to determine anti-oxidant enzyme activities. GPx activities were determined by commercialized test kits (Nanjing Jiancheng Bioengineering Institute, Nanjing, China) according to the manufacturer's protocol. GPx activity is expressed as U/mg protein. One unit (U) is defined as the amount of GPx that oxidizes 1 μmol L^−1^ of GSH per minute in the reaction system.

### 2.5 Transmission electron microscopy

Electron microscopy was conducted at the Center for Testing & Analysis Center in Nanjing Medical University. Small liver blocks were excised from the left lobe; a 1-mm intestinal segment from the 3-cm segment starting 1 cm distal to the pyloric valve was isolated and immediately fixed at 4 °C in a solution containing 4.0% (wt/vol) paraformaldehyde and 2.0% (wt/vol) glutaraldehyde in 0.1 M phosphate (pH 7.2) buffer for TEM analysis. The specimens were then postfixed in 1.0% osmium tetroxide, dehydrated in ethanol, and embedded in resin for sectioning. Sections were cut and contrast-stained with lead citrate and uranyl. The Advanced Microscopy Facility at the University of Virginia performed fixation, embedding and sectioning of the specimens and a JEOL 1010 or Tecnai 12 transmission electron microscope was used for high-resolution digital images. Mitochondrial lengths were measured on the tissue images using ImageJ (NIH). The percentage of mitochondria less than 500 nm in length was calculated.

### 2.6 Saponin-permeabilized tissues and ATP measurements

The production rate of ATP in the total mitochondrial population was performed using in situ fresh saponin-permeabilized fibers according to a previously described protocol [26]. Briefly, small pieces (2–5 mg) of liver and proximal intestine tissues were obtained from WT and *Per1*^*−/−*^ mice at ZT1 or ZT13 respectively and then permeabilized with 100 μg/mL saponin at 4 °C in buffer A containing (in mmol/L) K_2_EGTA 7.23, K_2_CaEGTA 2.77, MgCl_2_ 6.56, imidazole 20, dithiothreitol 0.5, K-MES 53.3, taurine 20, Na_2_ATP 5.3, PCr 15, and KH_2_PO_4_ 3, pH 7.1 adjusted at 25 °C. The fibers were then washed twice for 10 min in buffer B containing (in mmol/L) K_2_EGTA 7.23, K_2_Ca EGTA 2.77, MgCl_2_ 1.38, imidazole 20, dithiothreitol 0.5, K-MES 100, taurine 20, KH_2_PO_4_ 3, and BSA 2 mg/m L, pH 7.1 at 25 °C. The respiratory rates of saponin-permeabilized tissues were determined with the same oxygen sensor probe used for the MVO_2_ measurements in 2 mL of buffer B at 25 °C with continuous stirring. Studies were performed with the substrates glutamate 5 and malate 2 (in mmol/L). Oxygen consumption rates were expressed as nmol of O_2_ · min^−1^ · mg dry fiber weight. Respiratory parameters were defined as follows. Basal respiration rates before the addition of ADP were defined at state 2. Maximally ADP (1 mmol/L)-stimulated respiration rates were defined as state 3, and respiration rates in the absence of ADP phosphorylation and measured in the presence of 1 μg/mL oligomycin were termed state 4. The ATP synthesis rates were determined under state 3 conditions by sampling the respiratory buffer every 10 s for 60 s immediately after addition of ADP [27]. Briefly, fresh saponin-permeabilized tissues were added to the respiration chamber with 1 ml of buffer B (37 °C in a water bath) and wait for 3 min until the chamber had become accustomed to the buffer. Then, ADP and succinate were added, and a timer started at the same time. A sample of the buffer was taken every 10 s for 1 min. The chamber was placed on a preweighed piece of foil and allowed to dry overnight. The ATP concentration was determined by HPLC as described previously [28,29].

### 2.7 Sample preparation and NMR spectroscopy

A total of 300 μL of serum sample was added with 300 μL D_2_O (0.2 mol L^−1^Na_2_HPO_4_, and 0.2 mol L^−1^ NaH_2_PO_4_, pH 7.4, containing 0.05% TSP). The samples were vortexed and centrifuged at 13,000 g for 10 min at 4 °C to remove insoluble material. The supernatants were then pipetted into 5 mm NMR tubes for ^1^H NMR spectral acquisition. Liver tissues were obtained from WT and *Per1*^*−/−*^ mice at ZT1 and ZT13. Liver samples (with a wet weight of 0.3 g) were homogenized immediately in 600 μL of ice-cold acetonitrile/H_2_O (1:1 v/v) buffer, vortexed and then centrifuged at 12,000×*g* for 10 min at 4 °C. The supernatant was frozen and lyophilized to dryness and stored at −80 °C until the NMR test. The NMR samples were prepared by dissolving dried extracts in 600 μl D_2_O.After centrifugation at 12,000×*g* for 10 min, the supernatant was transferred to a 5 mm NMR tube for ^1^H NMR analysis. ^1^H NMR spectra of the samples were recorded on a Bruker AV 500 MHz spectrometer at 298 K. For liver tissue samples, modified nuclear Overhauser enhancement spectroscopy with a pre-saturation (NOESYPR) pulse sequence (relaxation delay-90°-μs-90°-tm-90°-acquire-FID) was used to suppress the residual water signal. Prior to the Fourier transformation, a line broadening of 0.3 Hz was applied to all spectra. An exponential weighting factor corresponding to a line broadening of 0.3 Hz was used to all acquired free induction decays prior to Fourier transformation and phase correction.

### 2.8 Spectral processing

The ^1^H NMR spectra of sample extracts were corrected for phase and baseline distortion and referenced manually to the TSP resonance at δ 0.00 using the TOPSPIN package (V3.0, Bruker Biospin, Germany). Spectral regions δ 0.50–9.50 were automatically binned using a dynamic adaptive binning approach with an equal width of 0.002 ppm. The noisy and residual water affected regions (4.40–5.70 ppm for serum, 4.65–5.25 ppm for liver) were removed. The remaining spectral data was normalized by probabilistic quotient normalization (PQN) prior to pattern recognition analysis [30].

### 2.9 NAD^+^ measurements

NAD^+^ levels were measured as previously described [31]. Briefly, frozen liver or small intestine tissues were homogenized in 1 M perchloric acid and neutralized in 3 M K_2_CO_3_ on ice. After centrifugation, the supernatant was mixed with Buffer A (50 mM K_2_PO_4_/KHPO_4_, pH 7.0) and loaded on to the column. The HPLC was run at a flow rate of 1 ml/min with 100% Buffer A from 0 to 5 min, a linear gradient to 95% Buffer A/5% Buffer B (100% methanol) from 5 to 6 min, 95% Buffer A/5% Buffer B from 6 to 11 min, a linear gradient to 85% Buffer A/15% Buffer B from 11 to 13 min, 85% Buffer A/15% Buffer B from 13 to 23 min, and a linear gradient to 100% Buffer A from 23 to 24 min. NAD^+^ eluted as a sharp peak at 15 min and was quantitated based on the peak area compared to a standard curve and normalized to tissue weight of frozen liver or small intestine tissues.

### 2.10 Construction of expression vectors

Mouse *Per1*, *Egfp* and *gpx1* were PCR amplified from the cDNA library, digested by the corresponding restrictive enzymes, and purified using agarose gel electrophoresis as described in Table S6. Briefly, fragments were amplified with PCR with the oligonucleotides described in Table S6, and then the PCR products were digested with restrictive enzymes respectively. At the same time, the plasmid was also digested by restrictive enzymes for the backbone. The fragments of double-digested products were subjected to the agarose gel electrophoresis and purified by a DNA purification kit (KarrotenScientific, Nanjing, China). The fragments and backbone were ligated by T4 ligase at 16 °C overnight to generate the new plasmids. The expression vectors were extracted and selected for sequencing (Sunnybio, Shanghai, China).

### 2.11 Preparation and purification of His-tagged proteins

Fragments PER proteins were expressed from a His-tag fusion protein containing amino acids 208 to 414 or amino acids 975 to 1290 of hPER1. The PCR-generated products of PER1 208–414 and PER1 975–1290 were inserted in the pET-29a-c(+) vectors with *Sal*I/*Not*I, respectively. The sequences used for PCR are shown in Table S6. These His-tagged proteins were purified as described previously [32]. Extracts were prepared from induced cells, and the soluble proteins were applied to individual Ni^2+^-agarose columns and washed with 20 mM Tris-HCl, pH 7.4, 40 mM imidazole, 0.5 M NaCl, 1 mM β-mercaptoethanol. His-tagged proteins were eluted with 200 mM imidazole in the same buffer, and Sodium dodecyl sulfate-polyacrylamide gel electrophoresis (SDS-PAGE) was used to analyze the polypeptides in the eluted fractions. Furthermore, HPLC was used to further ensure the purity is more than 95%, and the purified proteins were stored at −80 °C.

### 2.12 Live cell fluorescence and immunofluorescence staining

NIH-3T3 cells were cultured as described above and the cells were imaged 24–36 h post transfection. Briefly, live NIH-3T3 cell samples were transfected with pCMV-Sport2 Per1-EGFP plasmid for 24–36 h and then washed three times with Opti-MEM. Next, we removed the media from the dish and add prewarmed (37 °C) staining solution containing MitoTracker® Red CMXRos (100 nm) for 25 min. After staining, the cells were washed three times with Opti-MEM and then fixed with 3.7% formaldehyde in complete growth medium at 37 °C for 15 min. After fixation, the cells were rinsed 5 times in PBS buffer. For IF, paraformaldehyde-fixed NIH-3T3 cell samples were sequentially incubated with primary antibodies anti-GPX1 (Abcam, Cat#ab22604, Cambridge, MA, USA), anti-PER1 (Abcam, Cat# ab3443, Cambridge, MA, USA) and Alexa Fluor®568-conjugated secondary antibodies (ThermoFisher, Cat#A-11011, Waltham, MA, USA). Then, samples were imaged using a Delta Vision OMX system (GE Healthcare), and processed using GE SoftWoRx Imaging Analysis software.

### 2.13 RNA extraction and quantitative real-time RT-PCR

Total RNA was extracted from the samples with TRIzol (KarrotenScientific, Nanjing, China) according to the manufacturer's instructions. The reverse transcription reaction was carried out using a reverse transcriptase kit according to the manufacturer's protocol. Real-time PCR was performed, and the products were detected using the ABI 7300 Detection System with SYBR Green dye. The expression level of *actin* was simultaneously quantified as an internal standard control. The sequences of all primers used for quantitative RT-PCR are shown in Table S6.

### 2.14 Peroxide assays

Tissues were homogenized at a 1/9 (w/v) dilution in cold saline in an ice bath. Suspensions were centrifuged at 12,000×*g* for 10 min at 4 °C and supernatants were collected in ice bath and then used to determine peroxide levels. Peroxide measurements were performed as described previously [33]. Briefly, 50 μl of test sample is added to 950 μl of FOX reagent (xylenol orange 100 μM; ammonium ferrous sulfate 250 μM; sorbitol 100 mM; H_2_SO_4_ 25 μM) or FOX reagent without ammonium ferrous sulfate as background, vortexed, and then incubated at room temperature for a minimum of 30 min, at which time color development is virtually complete. The absorbance was read at 560 nm after removal of any flocculated material by centrifugation. The signal is read against a peroxide standard.

### 2.15 Quantitation of mtDNA

To determine the mitochondrial DNA content in livers and small intestines, DNA was extracted using a DNeasy kit (Qiagen), and quantitated by real-time PCR analysis using the ABI 7300 Detection System with SYBR Green dye (Toyobo, Osaka, Japan). Two specific primers for mitochondrial DNA were described previously [34]. The primer sequences were described in Table S6. Actin DNA was used for normalization as a genomic loading control.

### 2.16 Western blot analysis

Proteins were extracted following the procedure described previously [35]. The proteins were separated by SDS-PAGE on 8–15% polyacrylamide gels and subsequently electrically transferred to a PVDF membrane. After blocking with 5% (w/v) BSA in TBST at room temperature for 1 h, the membranes were then incubated with appropriate specific primary antibodies: anti-GPX1 (1:2000, Abcam, Cat#ab108427, Cambridge, MA, USA); anti-HA tag (1:1000, Cell Signaling, Cat# 3724, Danvers, MA, USA); anti-PER1 (1:200; Abcam, Cat# ab3443, Cambridge, MA, USA); anti-β-actin (1:1000, Bioworld, Cat# AP0060, MN, USA) at 4 °C overnight, followed by incubation with an HRP-conjugated secondary antibody (1:10000 Beyotime, Cat#A0208, Shanghai, China). Detection was performed using an enhanced chemiluminescence kit (Thermo Scientific, Hudson, NH, USA).

### 2.17 Co-Immunoprecipitations

Co-immunoprecipitation was performed as described previously with slight modifications [36]. Briefly from in vivo experiments, fresh livers were homogenized and lysed with a solution containing 10 mM Tris-HCl (pH 8.0), 420 mM NaCl, 1 mM EDTA and 0.5% NP-40 with protease inhibitor cocktail (Boster Biological Technology Ltd). Furthermore in vitro, NIH 3T3 cells were transfected with the HA-Per1 plasmids using Lipofectamine 2000 Transfection Reagent in 10-cm dishes and then lysed as described above. To prepare the immunoprecipitates, we incubated the lysates with specific antibodies overnight at 4 °C followed by incubation with Protein G-Sepharose 4B. The immunoprecipitates were washed five times with wash buffer containing 10 mM Tris-HCl (pH 8.0), 100 mM NaCl, 1 mM EDTA, 0.5% NP-40 and 0.5% Triton X-100 and subsequently boiled in SDS-PAGE loading buffer. Antibodies against GPX1, HA-tag, and PER1 were performed for co-immunoprecipitation. The proteins were analyzed by western blotting as described above.

### 2.18 Molecular docking of PER1 and GPX1 by Rosetta

Protein docking of GPX1 (PDB 2f8a) and PER1 (PDB 4dj2) was performed by the ZDOCK online server, using the default parameters. The resulting docking complex with the highest score was chosen as the final solution and subjected to molecular dynamics simulation (MD) for further optimization. MD was performed using the sander module implemented in the Amber 16 suite with the ff14SB force field. The simulation system was immersed in a truncated octahedral box of TIP3P explicit water, extended 10 Å outside the protein on all sides. To start the MD simulation, the initial structure of the docking complex was relaxed to minimize energy during 10,000 minimization steps (5000 steepest descent steps, SD, and 5000 conjugate-gradient steps, CG). After that, the system was gradually heated in the NVT ensemble from 0 to 300 K over 50 ps, followed by 50 ps of NPT simulation at 300 K and 1 atm pressure using the Langevin dynamics algorithm. After equilibration, a 20-ns production MD simulation was performed. The molecule coordinates of the system were saved every 10 ps? All processing and analysis of the trajectory data were performed using CPPTRAJ programs. The MD trajectories were applied to calculate the binding free energy of 4dj2 to 2f8a using the MM/GBSA module implemented in AMBER16. The binding free energy decomposition and ala-scanning were also performed using the corresponding modules implemented in the MMPBSA. py script.

### 2.19 Multivariate data analysis

Multivariate data analysis was performed by a suite of scripts developed in-house running in R software. PCA is an exploratory unsupervised method to maximize the separation by providing model-free approaches for determining the latent or intrinsic information in the dataset. However, no clustering was observed when variables were not selected. OSC-PLS-DA, which is a supervised method, was used to maximize the covariance between the measured data (peak intensities in NMR spectra) and the response variable (predictive classifications) [37]. All OSC-PLS-DA models were validated by a repeated two-fold cross-validation method and permutation test. The parameters of R^2^ and Q^2^ represented the goodness of fit and the predictive ability of the models, respectively [38].

### 2.20 Statistical analysis

All data are expressed as the means ± SEM. The statistical analysis of the results was performed using GraphPad Prism 8 software (San Diego, CA, USA). Two-sided, student's t-test was employed for studies involving two independent groups. One-way or two-way analysis of variance models with test for interaction between two factors were employed for multiple group comparisons with Turkey's P-value adjustment for multiple pairwise comparisons. Significance was set as *p < 0.05.

## 3 Results

### 3.1 Mice lacking *Per1* are unable to maintain energy metabolism rhythm

**Fig. 1:**
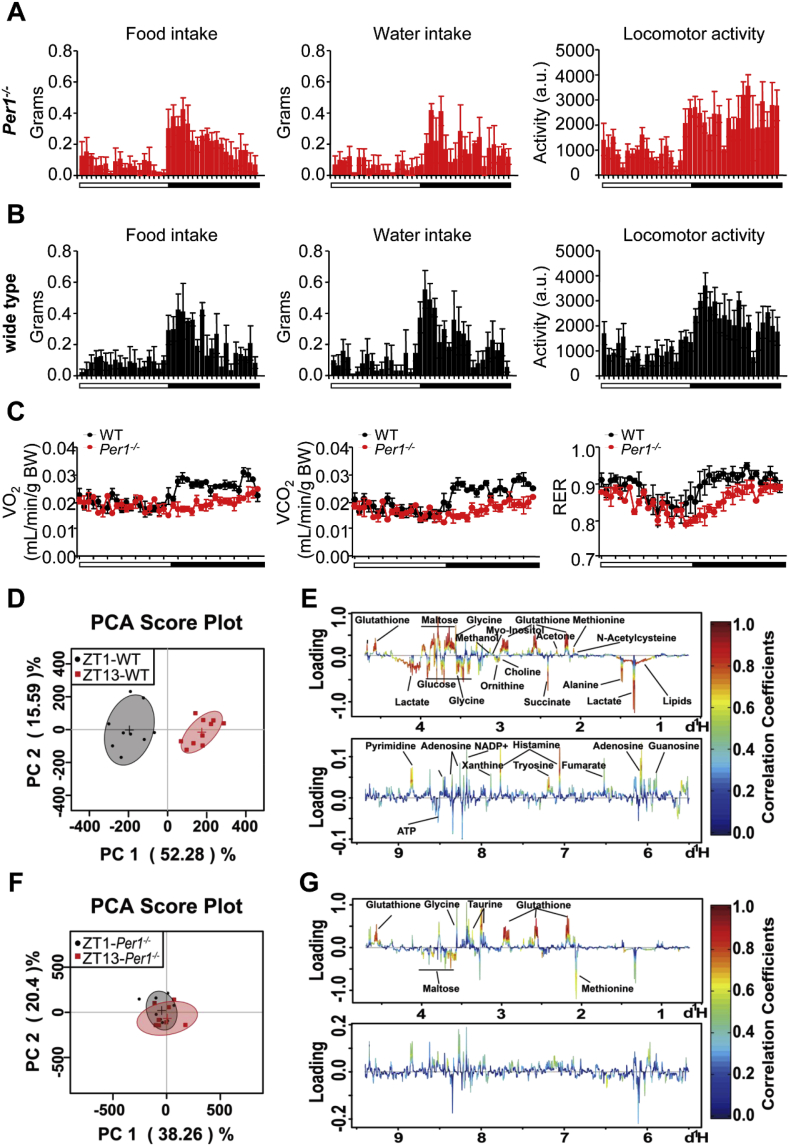
***Per1* Deficiency Uncouples Daily Energy Metabolic Rhythm from Behavior Oscillations**. A, B) Analysis of feeding behavior (left), water intake (middle), and locomotor activity (right) of *Per1*^−/−^ (A) and WT (B) mice (n = 4 per group) fed ad libitum. Food consumption, water intake, and voluntary locomotor activity were recorded using metabolic cages. Locomotor activity is presented in arbitrary units (a.u.). C) Oxygen consumption (left), carbon dioxide production (middle) and respiratory exchange ratio (right) were measured for 24 h (n = 4). Male WT and *Per1*^−/−^ mice fed a ND at 8–10 weeks old were acclimated to the chambers for 4 days and recordings were performed for 2 days, yielding one full 24-h periods of data using metabolic cages. D, E) PCA scores plot (D) and color-coded loading plots (E) of OSC-PLS-DA for liver extracts obtained from WT mice (n = 9–10/time point, 8 weeks old). F, G) PCA scores plot (F) and color-coded loading plots (G) of OSC-PLS-DA for liver extracts obtained from *Per1*^*−/−*^ mice (n = 8/time point, 8 weeks old). Metabolites that contributed to group separation were then visualized and color-coded according to the absolute correlation coefficient of each variable with each group. Color coding is in accordance with the fold change in metabolites, where red indicates a significant change. Normalized values are shown in Table S1. Throughout, all data were expressed as the mean ± SEM and male mice for this experiment were maintained on standard chow. (For interpretation of the references to color in this figure legend, the reader is referred to the Web version of this article.)

To examine how circadian clock anticipate daily fluctuations in energy demand and nutrient availability, we monitored daily food (also water) intake, voluntary locomotor activity and oxygen consumption in mice using metabolic cages. In agreement with previous reports, *Per1*-deficient (*Per1*^*−/−*^) mice (Fig. 1A) exhibited similar diurnal feeding rhythms and consumed equal food during the light phase or dark phase compared to WT mice (Fig. 1B). Unexpectedly, daily rhythm in body oxygen consumption and carbon dioxide production were impaired in *Per1*^*−/−*^ mice compared to WT mice (Fig. 1C, left, middle), and lower respiratory exchange ratio occurred throughout day and night in *Per1*^*−/−*^ mice (Fig. 1C, right), indicating *Per1*^*−/−*^ mice seem to uncouple energy metabolic rhythm from feeding and active oscillations. To clarify this observation, we carried out a ^1^H NMR-based metabolomics analysis for metabolites change in the liver and plasma of WT, and *Per1*^*−/−*^ mice. Mice were killed at ZT1 during day time and ZT13 during night time. Typical ^1^H NMR spectra showed rich compositional information of 40 metabolites for the liver extracts (Fig. S1) and metabolites were identified with their ^1^H resonances assigned, and the detailed proton chemical shifts and their multiplicity are listed in Table S1. The OSC-PLS-DA score plot analysis showed a clear separation between samples obtained from ZT1 and ZT13 in WT livers (Fig. 1D and E); and an overlapping was achieved between samples obtained from ZT1 and ZT13 in *Per1*^*−/−*^ mice (Fig. 1F and G). Similar pattern was observed in the plasma obtained from WT and *Per1*^*−/−*^ mice, respectively (Figs. S2A–S2E; Table S2). Together, these observations suggest circadian *Per1* is a potential intermediator for coupling metabolic rhythm with behavior oscillations in mammals.

### 3.2 *Per1* deficiency impairs daily mitochondrial dynamics in the peripheral organs

**Fig. 2:**
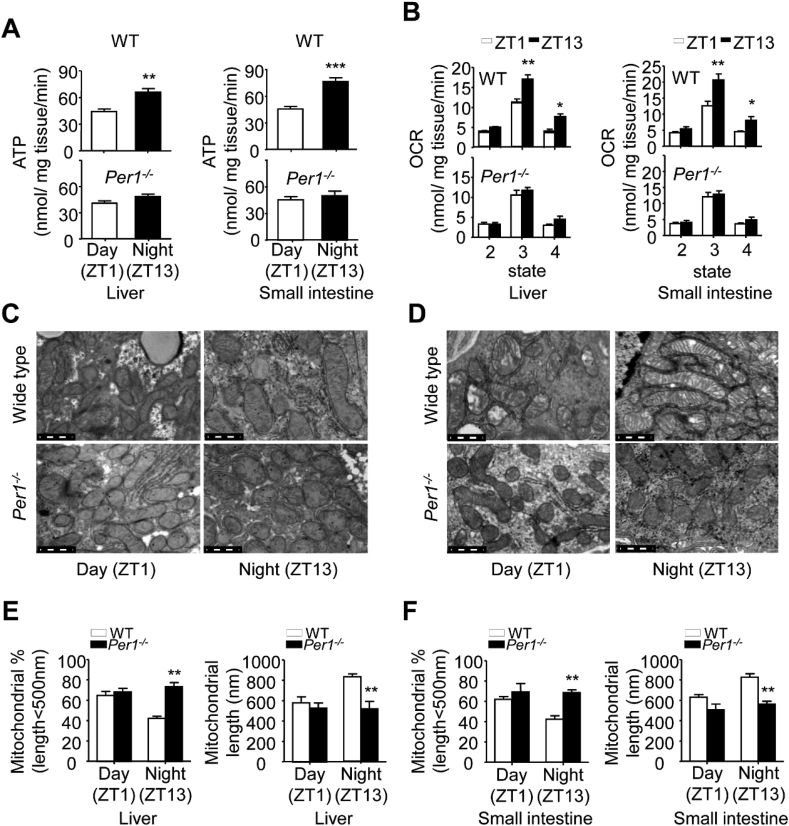
***Per1* Deficiency Impairs Daily Mitochondrial Morphology and Function in the Liver and Intestine**. A) The production rate of mitochondrial ATP in fresh livers (left) and intestine samples (right) of WT (top) and *Per1*^*−/−*^ (bottom) mice (6–8 weeks old) collected at ZT1 and ZT13, respectively (n = 4, 6–8 weeks old, **p < 0.01, ***p < 0.001, night versus day group). B) The OCR of mitochondria in fresh livers (left) and intestine samples (right) of WT (top) and *Per1*^*−/−*^ (bottom) mice (6–8 weeks old) collected at ZT1 and ZT13, respectively (n = 4, 6–8 weeks old, *p < 0.05, **p < 0.01, night versus day group). State 2 indicates respiration in the absence of ADP; state 3, ADP (1 mmol/L)-stimulated respiration; state 4, oligomycin (1 g/mL)-inhibited respiration. C, D) Representative TEM images of liver samples (C) and proximal intestine sections (D) from WT and *Per1*^*−/−*^ mice (n = 3, 8 weeks old) at ZT1 and ZT13, respectively, Scale bar, 0.5 μm. E, F) The percentage of mitochondrial less than 500 nm in length (left) and total mitochondrial lengths (right) calculated from EM images of liver samples (E) and proximal intestine sections (F) from WT and *Per1*^*−/−*^ mice at ZT1 and ZT13, respectively. Throughout, all data were expressed as the mean ± SEM, **p < 0.01, *Per1*^*−/−*^ versus WT group. Analyses were performed using two-way ANOVA for A, B, E, F. Male mice for all experiments were maintained on standard chow.

To show that impaired daily metabolic rhythm in *Per1*^*−/−*^ mice is involved in mitochondrial function, an HPLC assay was undertaken to measure mitochondrial ATP production efficiency via the released ATP from fresh tissues. We found that fresh liver and intestine tissues obtained from WT mice displayed a day-night difference in mitochondrial ATP production with lower efficiency at ZT1 and higher at ZT13 (Fig. 2A, top); The liver and intestine tissues obtained from *Per1*^*−/−*^ mice showed lower efficiency at ZT13 compared to that in WT mice in mitochondrial ATP production, and no diurnal pattern between ZT1 and ZT13 (Fig. 2A, bottom). Moreover, an oxygen consumption rate (OCR) analysis revealed that *Per1* deficiency impaired mitochondrial respiratory efficiency in fresh liver and intestine tissues (Fig. 2B). However, there was no difference of total mitochondrial DNA copy numbers between day and night in WT and *Per1*^*−/−*^ mice (Figs. S3A and S3B). Then, we examined whether circadian *Per1* directly impaired the transcription of some key target genes involved in mitochondrial dynamics, and obvious difference was observed in the liver and intestine tissues between genotypes (Figs. S3C and S3D; Table S3). Interestingly, while the ultrastructure of mitochondria in liver obviously displayed reproductive variation, and the geometry of mitochondria became significantly shorter and rounder at ZT1, displaying particular disarrangement and distortion of cristae compared to that at ZT13 in WT mice (Fig. 2C, top), no obviously diurnal variation of geometry of mitochondria could be detected in *Per1*^*−/−*^ livers (Fig. 2C, bottom). Similar pattern was observed in *Per1*^*−/−*^ intestines (Fig. 2D). Quantitative parameter for the changes in mitochondrial morphology confirmed these observations from representative images (Fig. 2E and F). Together, these results suggest that *Per1* deficiency impairs rhythmic regulation of mitochondrial function.

### 3.3 *Per1* deficiency deregulates daily shift on cellular GPx-related ROS fluctuations

**Fig. 3:**
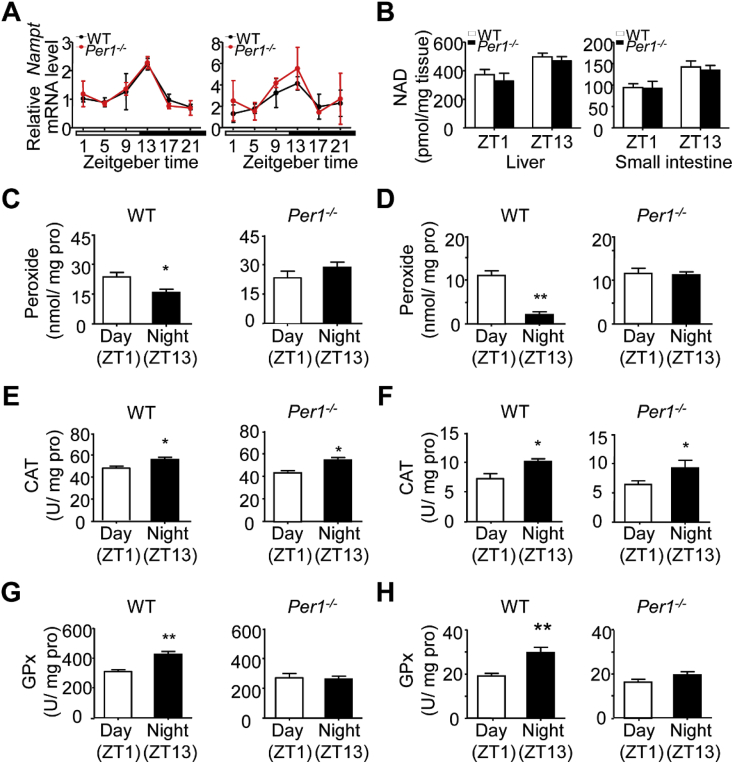
***Per1* Increases the Enzymatic Activity of GPx to Scavenge ROS**. A) Diurnal expression levels of *Nampt* in WT (black) and *Per1*^*−/−*^(red) mice livers (left) and small intestines (right) determined by quantitative real-time RT-PCR (n = 4/time point/genotype). The white and black bar represents day and night, respectively. No significant difference was observed between WT and *Per1*^*−/−*^ mice. B) NAD^+^ levels in WT and *Per1*^*−/−*^ mice livers (left) and small intestines (right) at ZT1 and 13 (n = 4–5/time point/genotype). No significant difference was observed between WT and *Per1*^*−/−*^ mice. C, D) Peroxide levels of the livers (C) and intestines (D) from WT (left) and *Per1*^*−/−*^ (right) mice, samples were collected at ZT1 and ZT13, respectively (n = 5, 8 weeks old, *p < 0.05, **p < 0.01, night versus day group). E, F) Catalase activity of the livers (E) and intestines (F) from WT (left) and *Per1*^*−/−*^ (right) mice, samples were collected at ZT1 and ZT3, respectively (n = 5, 8 weeks old, *p < 0.05, **p < 0.01, night versus day group). G, H) GPX activity of the livers (G) and intestines (H) from WT (left) and *Per1*^*−/−*^ (right) mice, samples were collected at ZT1 and ZT13, respectively (n = 5, 8 weeks old, *p < 0.05, **p < 0.01, night versus day group). Throughout, all data were expressed as mean ± SEM, and male mice for all experiments were maintained on standard chow. Analyses were performed using two-tailed Student's t-test for C–H, two-way ANOVA for B. (For interpretation of the references to color in this figure legend, the reader is referred to the Web version of this article.)

To determine whether the changes of mitochondrial morphology and function in *Per1*^*−/−*^ mice is driven by nicotinamide adenine dinucleotide (NAD^+^), a central node in coupling circadian and metabolic cycles [39], we monitored the expression profile of nicotinamide phosphoribosyltransferase (NAMPT) and measured NAD^+^ level in livers and intestines of both genotyping mice. The expression of NAMPT in liver and intestine of WT mice showed a robust circadian rhythm by quantitative RT-PCR analysis, and the expression curve of *Per1*^*−/−*^ mice remained the similar oscillation to WT mice (Fig. 3A). HPLC analysis revealed that there was also no difference in the NAD^+^ level of the liver and small intestine between *Per1*^*−/−*^ and WT mice (Fig. 3B), excluding a possible role of NAD^+^ in *Per1* regulating mitochondrial morphology and function. We then considered whether circadian *Per1* regulates reactive oxygen species (ROS) fluctuation generating a special intracellular environment for shift of the geometry of mitochondria. Proxide emerged as major redox metabolite operative in redox sensing, signaling and redox regulation [40]. The level of peroxide did have a robust diurnal pattern with a higher level at ZT1 and lower at ZT13 in WT liver (Fig. 3C, left). In contrast, liver samples obtained from *Per1*^*−/−*^ mice preserved higher peroxide levels compared to WT mice, and had no apparent diurnal variation between ZT1 and ZT13 (Fig. 3C, right). *Per1*^*−/−*^ intestines also showed higher level of peroxide than WT intestines (Fig. 3D). Next, we measured the activities of two hydrolase for peroxide, namely, catalase and glutathione peroxidase (GPx), respectively. A comparative analysis revealed that the activities of catalase and GPx had a strong diurnal pattern both in livers (Fig. 3E and G, left) and intestines (Fig. 3F and H, left), with higher activities at ZT13 than at ZT1 in WT mice. In the livers of *Per1*^*−/−*^ mice, while catalase still preserved a robust diurnal pattern like WT liver (Fig. 3E, right), the activity of GPx was much lower than that of WT mice, and exhibited no significant difference in liver extracts obtained from ZT1 and ZT13 (Fig. 3G, right). The profiles of GPx and ROS fluctuation in the intestines were similar to that observed in livers (Fig. 3F and H, right). Thus, *Per1* deficiency caused to impair GPX and ROS rhythm with low GPx activity and high ROS level in the peripheral organs. These observations indicate a potential link between circadian *Per1* and GPx activity.

### 3.4 PER1 modulates diurnal GPx activity irrelevant to transcriptional regulation

**Fig. 4:**
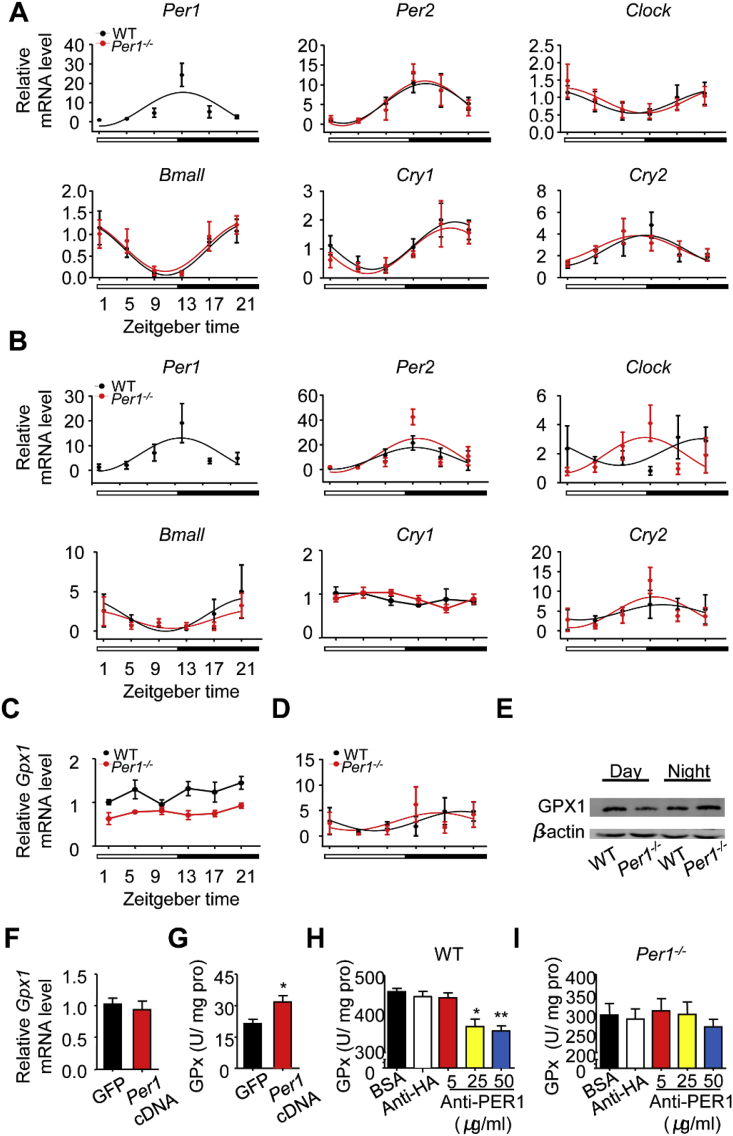
**PER1 Enhances Gpx Activity through Non-transcriptional Regulation**. A, B) The Expression Profiles of Clock Genes in (A) livers and (B) intestines between WT and *Per1*^*−/−*^ mice. Quantitative RT-PCR were performed for analyzing mRNA level in WT (black) and *Per1*^*−/−*^ (red) mice at indicated times (n = 4/time point/genotype). The white and black bar represents day and night, respectively. No significant differences in these genes' expressions were observed between genotypes. All data were analyzed in detailed Table S4. C, D) Real-time PCR analysis of diurnal mRNA expression of Gpx1 in WT and *Per1*^*−/−*^ livers (C) and intestines (D) (n = 4/time point/genotype). The white and black bar represents day and night, respectively. E) Western blot analysis of GPX1 protein level in the livers. Samples were respectively collected at ZT1 and ZT13 between genotypes and pooled samples (n = 3/time point/genotype) were used for each time point. β-actin was used as internal control. F) The mRNA levels of Gpx1 in NIH-3T3 cell line at 48 h after cell were transfected with Per1 cDNA (n = 6 per group). G) The GPx activity in the NIH-3T3 cell line at 48 h after the cells were transfected with Per1 cDNA (n = 6, *p < 0.05, Per1 cDNA versus GFP group). H, I) The GPx activities were measured in fresh liver extracts from WT (H) and *Per1*^*−/−*^ (I) mice when various concentrations of PER1 antibody were present. HA antibody (50 μg/ml) as a negative control (n = 4 per group, *p < 0.05, **p < 0.01, compared with control group). Throughout, all data were expressed as the mean ± SEM and male mice for all experiments were maintained on standard chow. Analyses were performed using two-tailed Student's t-test for F, G and one-way ANOVA for H, I. (For interpretation of the references to color in this figure legend, the reader is referred to the Web version of this article.)

To validate the direct regulation of *Per1* on GPx activity, we first examined whether the loss of *Per1* influences *Gpx1* and other clock genes transcription. *Per1*^*−/−*^ mice still preserved robust rhythmic expressions of *Clock*, *Bmal1*, *Per2*, *Cry1* and *Cry2* over a 24-h period in both the livers and intestines (Fig. 4A and B, Table S4), the transcription level of *Gpx1* had no obvious shift over 24 h both in either *Per1*^*−/−*^ or WT mice (Fig. 4C and D). Although GPx activity has a strong diurnal variation pattern, the GPX1 protein level has not obvious diurnal variation in WT or *Per1*^*−/−*^ mice (Fig. 4E). Delivering of *Per1* into NIH3T3 cells did not lead to changes in *Gpx1* gene levels (Fig. 4F), but resulted in a significant increase in GPx activity (Fig. 4G). The level of GPX protein was not directly related to its enzyme activity [41]. Surprisingly, PER1 antibody inhibited GPx activity in a dose-dependent manner (Fig. 4H and I). These observations indicate that PER1 protein could directly regulate GPx activity.

### 3.5 The PER1 interaction with GPX1 forms a complex

**Fig. 5:**
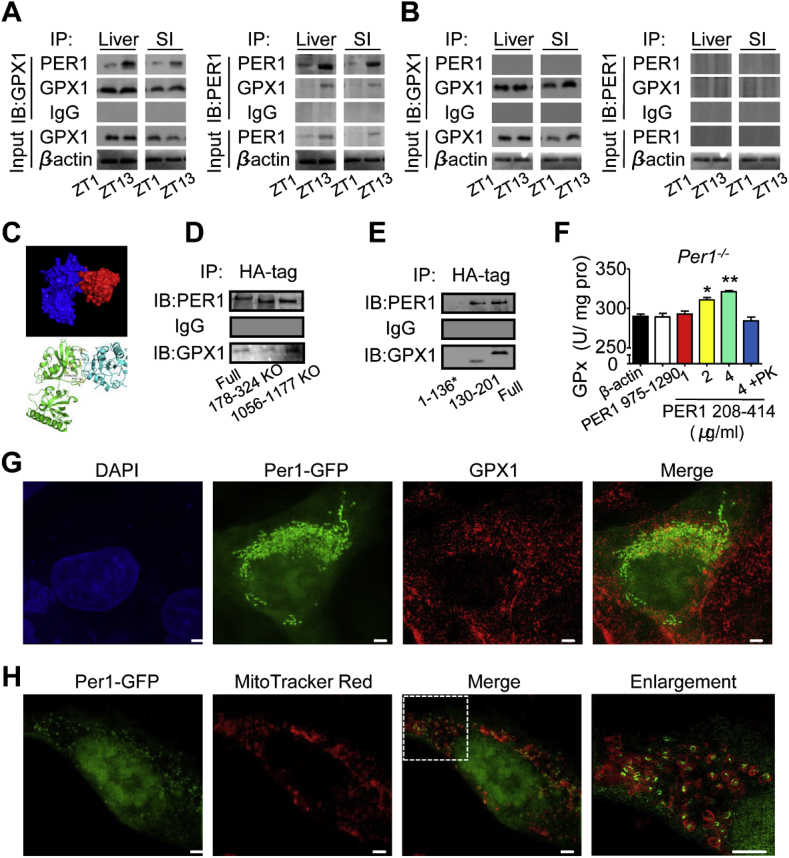
**PER1 Binding to GPX1 Mediates Diurnal GPx Activity**. A, B) Representative co-immunoprecipitation results of PER1 and GPX1 interaction from cytoplasmic extracts from WT (A) and *Per1*^*−/−*^ (B) mouse liver or small intestine at ZT1 or ZT13. β-actin as an input control. C) Molecular docking of GPX1 to PER1 and the backbone of PER1 and GPX-1 are shown in blue and red (C, top), respectively. Location of key residues was identified by mutagenesis (C, bottom). D-E) Representative immunoprecipitation analysis of PER1 and GPX1 interaction from lysates from NIH-3T3 cells. Lysates from NIH-3T3 cells expressing HA-tagged PER1 or mutant forms of PER1 (178–324 aa deletion and 1056–1177 aa deletion as CK) were immunoprecipitated with an anti-HA antibody (D). Lysates from NIH-3T3 cells coexpressing HA-tagged fragments of GPX1 and a vector expressing PER1 were immunoprecipitated with an anti-HA antibody (E). *GPX1 residues 1–136 are not detected because the UGA stop codon terminates translation in advance while recoded as selenocysteine (46 aa) in full-length conditions. F) In vitro a supplement of the fragment PER1 208–414 increases Gpx activity in *Per1*^*−/−*^ mice (n = 4 per group, *p < 0.05, **p < 0.01 versus β-actin group). β-actin, fragment PER1975-1290 (4 μg/ml) and Proteinase K (PK) digested the fragment PER1 208–414(4 μg/ml) products were performed as negative controls. G, H) Representative immunofluorescence images of GPX1 (Alexa Fluor 568-labeled) and Per1-GFP in NIH-3T3 cells (G). MitoTracker Red CMXRos-labeled mitochondria and Per1-GFP staining in NIH-3T3 cells (H). Images were obtained using a Delta Vision OMX system (GE Healthcare) and processed using GE SoftWoRx Imaging Analysis software. Bars indicate 1 μm in length. Throughout, male mice for all experiments were maintained on standard chow. Data are presented as the mean ± SEM and analyses were performed using one-way ANOVA for F. (For interpretation of the references to color in this figure legend, the reader is referred to the Web version of this article.)

The mitochondrial GPX1 is the product of the *Gpx1* gene. To investigate whether PER1 and GPX1 proteins can form a complex, a co-immunoprecipitation (co-IP) of PER1 and GPX1 was performed. As shown in Fig. 5A and B, PER1 directly bound to GPX1 and that the binding efficiency exhibited obvious diurnal changes in the liver and intestine of WT mice but not in *Per1*^*−/−*^ mice, suggesting that PER1 binds GPX1 to regulate GPx activity. A Rosetta-based molecular docking analysis revealed the putative position of the GPX1 complex with PER1 (Fig. 5C, and Table S5). Our results showed that mutating PER1178-324 caused a reduction in binding affinity (Fig. 5D), and amino acids 130–201 of GPX1 interacted strongly with PER1 (Fig. 5E). Next, to further illustrate whether PER1 structurally directly enhances GPx activity, we generated two fragments of the PER1 protein, referred to here as PER1 208–414 and PER1 975–1290. As expected, the PER1 208–414 binding site increased Gpx activity in a dose-dependent manner (Fig. 5F), but PER1 975–1290 had no effect on Gpx activity. Finally, we induced Per1-GFP by transfection to investigate PER1-mitochondria and PER1-GPX1 colocalization. Two-color confocal images of cells revealed that PER1 localized in both the nucleus and cytoplasm (Fig. 5G). Under the conditions tested, substantially larger amounts of PER1 were colocalized with GPX1 (Fig. 5G) and around mitochondria (Fig. 5H). Together, these data suggest that PER1 interaction with GPX1 forms a complex.

### 3.6 Fasting stress specifically elevated *Per1* expression in peripheral organs for maintaining mitochondrial function under oxidation stress

**Fig. 6:**
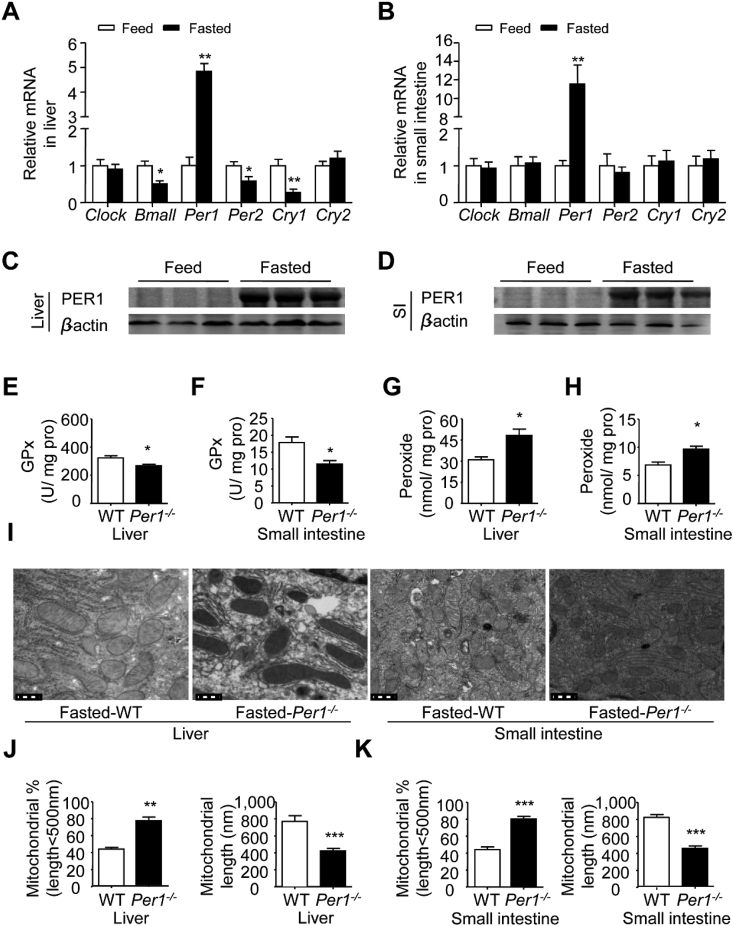
***Per1* Protects Mitochondrial Function from Fasting-Induced Oxidative Stress**. A, B) The expression profiles of clock genes in the livers (A) and intestines (B) of WT mice in fasting stress compared to those in feeding at ZT0. Quantitative RT-PCR was performed for analyzing mRNA level in fed (white) and fasted (black) WT mice (n = 5 per group, *p < 0.05, **p < 0.01 compared with fed group). C, D) Western blot analysis of PER1 protein level in livers (C) and intestines (D) of WT mice in fasting stress compared to those in feeding at ZT1 (n = 3). β-actin was used as internal control. E, F) GPx activity of the livers (E) and intestines (F) from WT and *Per1*^*−/−*^ mice were measured under fasting condition (n = 5 per group, *p < 0.05 compared with WT group). G, H) Peroxide levels of the livers (G) and intestines (H) from WT and *Per1*^*−/−*^ mice were measured under fasting condition (n = 5 per group, *p < 0.05 compared with WT group). I) TEM analysis of the geometry of mitochondria in livers and intestines from fasting WT and *Per1*^*−/−*^ mice at ZT0, n = 3. Bar = 0.5 μm. J, K) The percentage of mitochondrial less than 500 nm in length (left) and total mitochondrial lengths (right) calculated from EM images in fasted WT and *Per1*^*−/−*^ mouse livers (J) and intestines (K) at ZT0 (n = 3, **p < 0.01, ***p < 0.001, *Per1*^*−/−*^ versus WT group). Throughout, all data were expressed as the mean ± SEM and male mice for all experiments were maintained on standard chow. Analyses were performed using two-tailed Student's t-test for A and B, E-H, J and K.

To farther investigate the potential role of *Per1* on mitochondrial function in energy stress, we compared the energy changes of mice fed ad libitum with that of mice subjected to metabolic stress. Energy stress was generated by food deprivation for 12 h from ZT12 to ZT24. Interestingly, WT mice undergoing fasting stress showed that only *Per1* mRNA level was at least 4–6 fold higher in livers and intestines compared to those in feeding condition (Fig. 6A and B), and other clock genes such as *Per2*, *Clock* and *Bmal1* showed no obvious or low expression pattern (Fig. 6A and B). Accordingly, PER1 protein was significantly increased in the livers and intestines in the fasted state (Fig. 6C and D). To clarify the role of *Per1* in fasting conditions, we measured the redox level and related enzyme activity in *Per1*^*−/−*^ mice under fasting stress. We found that GPx activity decreased and peroxide levels increased both in the livers and intestines of fasted *Per1*^*−/−*^ mice (Fig. 6E–H). Moreover, our TEM analysis revealed that the geometry of mitochondria was dramatically changed (Fig. 6I) in that they had a significantly longer shape and apparent arrangement of cristae in fasting WT mice at ZT1; in *Per1*^*−/−*^ mice, fasting stress changed the geometry of mitochondria both in the intestines and livers, such that they displayed much more severe disarrangement, distortion of cristae and undistinguished ultrastructure (Fig. 6J and K). In conclusion, these observations suggest that *Per1* play an irreplaceable role in protecting mitochondrial function from oxidative stress.

## 4 Discussion

The main conclusion from our results is that circadian *Per1* is a major driven factor in regulating diurnal mitochondrial dynamics under oxidation stress. Oscillatory PER1 interaction with GPX1 increases mitochondrial ROS clearance and causes daily variations of mitochondrial respiratory efficiency involved in energy homeostasis. The gene *Per1* is thought to be part of a negative feedback loop generating circadian oscillations in cellular functions in the nucleus [42]. Although *Per1*-deficient mice display a shorter circadian period with reduced precision and stability in constant darkness [43], these mice appear to have normal rhythms in feeding and locomotor activity in light/dark (12/12) cycles. In general, oxygen consumption exhibits daily oscillations in experimental rodent models purely during the active phase [1,3]. Time-of-day-dependent oscillations in metabolic rhythms are closely associated the fluctuations in daily feeding and locomotor behaviors [2,44,45]. Our studies showed that loss of *Per1* in mice disrupt the rhythmic variation of oxygen consumption and metabolites level, which reveals an irreplaceable role of *Per1* on coupling metabolic rhythm with feeding behavior oscillation.

Accumulating evidence supports diurnal oscillations in mitochondrial energy metabolism and associated oxidative pathways in peripheral tissues [46,47]. Mitochondria provide the majority of cellular energy needed for metabolic processes through oxidative phosphorylation. Several nuclear-encoded mitochondrial proteins involved in oxidative phosphorylation display circadian oscillations in the mRNA levels [8]. Other key bioenergetic parameters including mitochondrial membrane potential, cytochrome *c* oxidase activity and coordination of mitochondrial Ca^2+^ handling, also show diurnal oscillations [48,49]. Although substantial evidence suggests that specific mitochondrial functions are circadian controlled [16], much less is known regarding mitochondrial bioenergetics under oxidation stress and the importance of the circadian clock in this vital process, suggesting there are several different levels of clock control over mitochondrial metabolism, including both transcriptional and post-transcriptional mechanisms. Our results presented here indicate that PER1 protein directly binds to GPX1 protein in cytoplasm and enhances its enzyme activity. Previous observation showed GPx activity, but not *Gpx1* RNA and protein levels, had a strong diurnal pattern, indicating GPx activity was not only dependent on protein level [50]. GPX1 is a crucial antioxidant enzyme involved in preventing the harmful accumulation of intracellular peroxide, which is present in mitochondrial and cytoplasm in all cells [[51], [52], [53], [54]]; and has been found to be more effective than catalase at removing intracellular peroxides (ROS) under many physiological conditions [55]. Genetic inactivation of *Gpx1* causes to increase oxidative stress and reduce mitochondrial ATP production [56], and circadian PER1 interaction with GPX1 creates a daily GPx activity rhythm and consequent ROS oscillation. It has been thought that ROS fluctuation causes daily variations of ultrastructure of mitochondria and its respiratory efficiency involved in energy productivity [13,[57], [58], [59], [60], [61]].

Our studies presented here provide compelling evidence that under oxidative stress, mitochondrial respiratory chain function and bioenergetics also are dependent on the molecular clock component PER1. Loss of *Per1* in mice severely weakened the ability of mitochondria to resist ROS under oxidation stress. In the absence of PER1, none of the other clock genes, including *mPer2*, *mCry1*, *mClock* and *mBmal1*, shows any obvious change in expression levels [62], providing a potential explanation for maintaining normal feeding and behavior rhythms in *Per1*-deficent mice. Fasting stress solely increases *Per1* expression, but not other clock components, suggesting an irreplaceable role of PER1 on response to oxidative stress. Loss of *Per1* causes highly visible abnormal changes in mitochondrial morphology and significant reduction in ATP production efficiency. Therefore, circadian PER1 synchronizes mitochondrial ATP production to meet fluctuations in cellular energy demands under oxidative stress.

A growing body of literature suggests that social stress, broadly defined as an individual's negative response to environmental pressure, is one contributor to the development metabolic syndrome both in adults and youths [63,64]. The circadian system must continuously adapt to and synchronize our physiology with the environment, and the *Per1* promoter has been proven to act as a sensor for multiple signaling molecules including protein kinases A and C (PKA, PKC) and cAMP responsive element binding protein (CREB), thereby integrating different physiological parameters for rapid adaptation to changing environmental conditions [65]. Thus, circadian *Per1* is a critical candidate in response to oxidative stress.

## Author contributions

Methodology, Q.S, Y.X.Y. and Z.Q.W.; Investigation, Q.S, Y.X.Y, X.Y, Y.G, Y.Z.; W.H.G, J.H.L. and X.X.; Writing-Original Draft, Q.S. and J.F.Z.; Writing-Review & Editing, S.M.W, D.W, J.S.W. and J.F.Z.; Resources, Z.Q.W, W.G. and J.F.Z.; Funding Acquisition, J.F.Z.; Conceptualization J.F.Z.

## Declaration of competing interest

The authors declare that they have no known competing financial interests or personal relationships that could have appeared to influence the work reported in this paper.

## References

[1] Alberts P., Johansson B.G., McArthur R.A. (2006). Characterization of energy expenditure in rodents by indirect calorimetry. Curr. Protoc. Neurosci..

[2] Bray M.S., Ratcliffe W.F., Grenett M.H., Brewer R.A., Gamble K.L., Young M.E. (2013). Quantitative analysis of light-phase restricted feeding reveals metabolic dyssynchrony in mice. Int. J. Obes..

[3] Pittendrigh C.S., Daan S. (1976). A functional analysis of circadian pacemakers in nocturnal rodents. J. Comp. Physiol..

[4] Scheer F.A., Hilton M.F., Mantzoros C.S., Shea S.A. (2009). Adverse metabolic and cardiovascular consequences of circadian misalignment. Proc. Natl. Acad. Sci. Unit. States Am..

[5] Sahar S., Sassone-Corsi P. (2012). Regulation of metabolism: the circadian clock dictates the time. Trends Endocrinol. Metabol..

[6] Nakahata Y., Sahar S., Astarita G., Kaluzova M., Sassone-Corsi P. (2009). Circadian control of the NAD+ salvage pathway by CLOCK-SIRT1. Science.

[7] Kaasik K., Lee C.C. (2004). Reciprocal regulation of haem biosynthesis and the circadian clock in mammals. Nature.

[8] Panda S., Antoch M.P., Miller B.H., Su A.I., Schook A.B., Straume M., Schultz P.G., Kay S.A., Takahashi J.S., Hogenesch J.B. (2002). Coordinated transcription of key pathways in the mouse by the circadian clock. Cell.

[9] Nikonova E.V., Vijayasarathy C., Zhang L., Cater J.R., Galante R.J., Ward S.E., Avadhani N.G., Pack A.I. (2005). Differences in activity of cytochrome C oxidase in brain between sleep and wakefulness. Sleep.

[10] Schibler U. (2005). The daily rhythms of genes, cells and organs: biological clocks and circadian timing in cells. EMBO Rep..

[11] Sakkou M., Wiedmer P., Anlag K., Hamm A., Seuntjens E., Ettwiller L., Tschöp M.H., Treier M. (2007). A role for brain-specific homeobox factor Bsx in the control of hyperphagia and locomotory behavior. Cell Metabol..

[12] Peek C.B., Ramsey K.M., Marcheva B., Bass J. (2012). Nutrient sensing and the circadian clock. Trends Endocrinol. Metabol..

[13] de Goede P., Wefers J., Brombacher E.C., Schrauwen P., Kalsbeek A. (2018). Circadian rhythms in mitochondrial respiration. J. Mol. Endocrinol..

[14] Forner F., Foster L.J., Campanaro S., Valle G., Mann M. (2006). Quantitative proteomic comparison of rat mitochondria from muscle, heart, and liver. Mol. Cell. Proteomics.

[15] Fernández-Vizarra E., Enríquez J.A., Pérez-Martos A., Montoya J., Fernández-Silva P. (2011). Tissue-specific differences in mitochondrial activity and biogenesis. Mitochondrion.

[16] Langmesser S., Albrecht U. (2006). Life time—circadian clocks, mitochondria and metabolism. Chronobiol. Int..

[17] Sardon Puig L., Valera-Alberni M., Canto C., Pillon N.J. (2018). Circadian rhythms and mitochondria: connecting the dots. Front. Genet..

[18] Woldt E., Sebti Y., Solt L.A., Duhem C., Lancel S., Eeckhoute J., Hesselink M.K., Paquet C., Delhaye S., Shin Y. (2013). Rev-erb-α modulates skeletal muscle oxidative capacity by regulating mitochondrial biogenesis and autophagy. Nat. Med..

[19] Peek C.B., Affinati A.H., Ramsey K.M., Kuo H.-Y., Yu W., Sena L.A., Ilkayeva O., Marcheva B., Kobayashi Y., Omura C. (2013). Circadian clock NAD+ cycle drives mitochondrial oxidative metabolism in mice. Science.

[20] Miwa S., Lawless C., Von Zglinicki T. (2008). Mitochondrial turnover in liver is fast in vivo and is accelerated by dietary restriction: application of a simple dynamic model. Aging Cell.

[21] Balaban R.S., Nemoto S., Finkel T. (2005). Mitochondria, oxidants, and aging. Cell.

[22] Cadenas E., Davies K.J. (2000). Mitochondrial free radical generation, oxidative stress, and aging. Free Radic. Biol. Med..

[23] Sies H., Cadenas E. (1985). Oxidative stress: damage to intact cells and organs. Philos. Trans. R. Soc. Lond. B Biol. Sci..

[24] Dröge W. (2002). Free radicals in the physiological control of cell function. Physiol. Rev..

[25] Zheng B., Larkin D.W., Albrecht U., Sun Z.S., Sage M., Eichele G., Lee C.C., Bradley A. (1999). The mPer2 gene encodes a functional component of the mammalian circadian clock. Nature.

[26] Veksler V.I., Kuznetsov A.V., Sharov V.G., Kapelko V.I., Saks V.A. (1987). Mitochondrial respiratory parameters in cardiac tissue: a novel method of assessment by using saponin-skinned fibers. Biochim. Biophys. Acta.

[27] Boudina S., Sena S., O'Neill B.T., Tathireddy P., Young M.E., Abel E.D. (2005). Reduced mitochondrial oxidative capacity and increased mitochondrial uncoupling impair myocardial energetics in obesity. Circulation.

[28] Knudsen T.B., Winters R.S., Otey S.K., Blackburn M.R., Airhart M.J., Church J.K., Skalko R.G. (1992). Effects of (R)-deoxycoformycin (pentostatin) on intrauterine nucleoside catabolism and embryo viability in the pregnant mouse. Teratology.

[29] Burkeen J.F., Womac A.D., Earnest D.J., Zoran M.J. (2011). Mitochondrial calcium signaling mediates rhythmic extracellular ATP accumulation in suprachiasmatic nucleus astrocytes. J. Neurosci..

[30] Dieterle F., Ross A., Schlotterbeck G., Senn H. (2006). Probabilistic quotient normalization as robust method to account for dilution of complex biological mixtures. Application in 1H NMR metabonomics. Anal. Chem..

[31] Ramsey K.M., Yoshino J., Brace C.S., Abrassart D., Kobayashi Y., Marcheva B., Hong H.K., Chong J.L., Buhr E.D., Lee C., Takahashi J.S., Imai S., Bass J. (2009). Circadian clock feedback cycle through NAMPT-mediated NAD+ biosynthesis. Science.

[32] Heath R.J., Rock C.O. (1996). Regulation of fatty acid elongation and initiation by acyl-acyl carrier protein in Escherichia coli. J. Biol. Chem..

[33] Wolff S.P. (1994). Ferrous ion oxidation in presence of ferric ion indicator xylenol orange for measurement of hydroperoxides. Methods Enzymol..

[34] Pua H.H., Guo J., Komatsu M., He Y.W. (2009). Autophagy is essential for mitochondrial clearance in mature T lymphocytes. J. Immunol..

[35] Wang T., Wang Z., Yang P., Xia L., Zhou M., Wang S., Du J., Zhang J. (2016). PER1 prevents excessive innate immune response during endotoxin-induced liver injury through regulation of macrophage recruitment in mice. Cell Death Dis..

[36] Pascual G., Fong A.L., Ogawa S., Gamliel A., Li A.C., Perissi V., Rose D.W., Willson T.M., Rosenfeld M.G., Glass C.K. (2005). A SUMOylation-dependent pathway mediates transrepression of inflammatory response genes by PPAR-gamma. Nature.

[37] Jung J.Y., Lee H.S., Kang D.G., Kim N.S., Cha M.H., Bang O.S., Ryu D.H., Hwang G.S. (2011). 1H-NMR-based metabolomics study of cerebral infarction. Stroke.

[38] Hochberg Y., Benjamini Y. (1990). More powerful procedures for multiple significance testing. Stat. Med..

[39] Peek C.B., Affinati A.H., Ramsey K.M., Kuo H.Y., Yu W., Sena L.A., Ilkayeva O., Marcheva B., Kobayashi Y., Omura C., Levine D.C., Bacsik D.J., Gius D., Newgard C.B., Goetzman E., Chandel N.S., Denu J.M., Mrksich M., Bass J. (2013). Circadian clock NAD+ cycle drives mitochondrial oxidative metabolism in mice. Science.

[40] Sies H. (2017). Hydrogen peroxide as a central redox signaling molecule in physiological oxidative stress: oxidative eustress. Redox Biology.

[41] Baker R.D., Baker S.S., LaRosa K., Whitney C., Newburger P.E. (1993). Selenium regulation of glutathione peroxidase in human hepatoma cell line Hep3B. Arch. Biochem. Biophys..

[42] Gekakis N., Staknis D., Nguyen H.B., Davis F.C., Wilsbacher L.D., King D.P., Takahashi J.S., Weitz C.J. (1998). Role of the CLOCK protein in the mammalian circadian mechanism. Science.

[43] Zheng B., Albrecht U., Kaasik K., Sage M., Lu W., Vaishnav S., Li Q., Sun Z.S., Eichele G., Bradley A. (2001). Nonredundant roles of the mPer1 and mPer2 genes in the mammalian circadian clock. Cell.

[44] Bailey S.M., Udoh U.S., Young M.E. (2014). Circadian regulation of metabolism. J. Endocrinol..

[45] Yang J., Wang Y., Garcia‐Roves P., Björnholm M., Fredholm B.B. (2010). Adenosine A3 receptors regulate heart rate, motor activity and body temperature. Acta Physiol..

[46] Young M.E., Razeghi P., Cedars A.M., Guthrie P.H., Taegtmeyer H. (2001). Intrinsic diurnal variations in cardiac metabolism and contractile function. Circ. Res..

[47] Bray M.S., Shaw C.A., Moore M.W., Garcia R.A., Zanquetta M.M., Durgan D.J., Jeong W.J., Tsai J.-Y., Bugger H., Zhang D. (2008). Disruption of the circadian clock within the cardiomyocyte influences myocardial contractile function, metabolism, and gene expression. Am. J. Physiol-Heart C.

[48] Isobe Y., Hida H., Nishino H. (2011). Circadian rhythm of metabolic oscillation in suprachiasmatic nucleus depends on the mitochondrial oxidation state, reflected by cytochrome C oxidase and lactate dehydrogenase. J. Neurosci. Res..

[49] Burkeen J.F., Womac A.D., Earnest D.J., Zoran M.J. (2011). Mitochondrial calcium signaling mediates rhythmic extracellular ATP accumulation in suprachiasmatic nucleus astrocytes. J. Neurosci..

[50] Baker R.D., Baker S.S., LaRosa K., Whitney C., Newburger P.E. (1993). Selenium regulation of glutathione peroxidase in human hepatoma cell line Hep3B. Arch. Biochem. Biophys..

[51] Orrenius S. (2007). Reactive oxygen species in mitochondria-mediated cell death. Drug Metab. Rev..

[52] Ott M., Gogvadze V., Orrenius S., Zhivotovsky B. (2007). Mitochondria, oxidative stress and cell death. Apoptosis.

[53] Esworthy R.S., Ho Y.-S., Chu F.-F. (1997). TheGpx1Gene encodes mitochondrial glutathione peroxidase in the mouse liver. Arch. Biochem. Biophys..

[54] Handy D.E., Lubos E., Yang Y., Galbraith J.D., Kelly N., Zhang Y.Y., Leopold J.A., Loscalzo J. (2009). Glutathione peroxidase-1 regulates mitochondrial function to modulate redox-dependent cellular responses. J. Biol. Chem..

[55] Casteilla L., Rigoulet M., Pénicaud L. (2001). Mitochondrial ROS metabolism: modulation by uncoupling proteins. IUBMB Life.

[56] Esposito L.A., Kokoszka J.E., Waymire K.G., Cottrell B., MacGregor G.R., Wallace D.C. (2000). Mitochondrial oxidative stress in mice lacking the glutathione peroxidase-1 gene. Free Radic. Biol. Med..

[57] Jacobi D., Liu S., Burkewitz K., Kory N., Knudsen N.H., Alexander R.K., Unluturk U., Li X., Kong X., Hyde A.L. (2015). Hepatic Bmal1 regulates rhythmic mitochondrial dynamics and promotes metabolic fitness. Cell Metabol..

[58] Cogliati S., Frezza C., Soriano M.E., Varanita T., Quintana-Cabrera R., Corrado M., Cipolat S., Costa V., Casarin A., Gomes L.C. (2013). Mitochondrial cristae shape determines respiratory chain supercomplexes assembly and respiratory efficiency. Cell.

[59] Sena L.A., Chandel N.S. (2012). Physiological roles of mitochondrial reactive oxygen species. Mol. Cell..

[60] Chen H., Chan D.C. (2004). Mitochondrial dynamics in mammals. Curr. Top. Dev. Biol..

[61] Campello S., Scorrano L. (2010). Mitochondrial shape changes: orchestrating cell pathophysiology. EMBO Rep..

[62] Cermakian N., Monaco L., Pando M.P., Dierich A., Sassone‐Corsi P. (2001). Altered behavioral rhythms and clock gene expression in mice with a targeted mutation in the Period1 gene. EMBO J..

[63] De Vriendt T., Moreno L.A., De Henauw S. (2009). Chronic stress and obesity in adolescents: scientific evidence and methodological issues for epidemiological research. Nutr. Metab. Cardiovasc. Dis..

[64] Wilson S.M., Sato A.F. (2014). Stress and paediatric obesity: what we know and where to go. Stress Health.

[65] Motzkus D., Maronde E., Grunenberg U., Lee C.C., Forssmann W.-G., Albrecht U. (2000). The human PER1 gene is transcriptionally regulated by multiple signaling pathways. FEBS Lett..

