# Supplementary material for: PER1 interaction with GPX1 regulates metabolic homeostasis under oxidative stress
Source: Redox Biol. 2020 Aug 26;37:101694. doi: 10.1016/j.redox.2020.101694 (PMC7484554; doi:10.1016/j.redox.2020.101694)
Supplement: Multimedia component 1 [file mmc1.docx]

**Supporting Information**

**
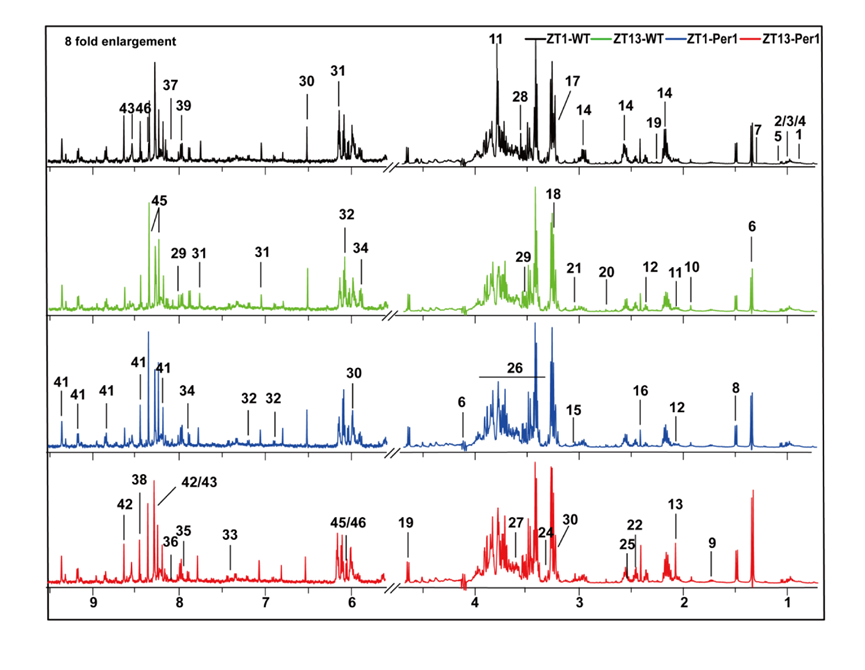
**

**Figure S1. OPLS-DA Analyses Based on ^1^H NMR Data from Livers Extracts of WT and *Per1^-/-^* mice.**

Typical 500 MHz ^1^H NMR spectra of liver extracts with metabolites assigned. 1. Cholate; 2/3/4. BCAAs; 5. Isobutyrate; 6. Lactate; 7. Lipids; 8. Alanine; 9. Lysine; 10. Acetate; 11. N-Acetylcysteine; 12. Glutamate; 13. Methionine; 14. Glutathione; 15.Creatine 16.Succinate; 17.Glutamine; 18.Sarcosine 19. Acetone; 20. Citrate; 21. Ornithine; 22. Choline; 23.Carnitine; 24. Myo-Inositol; 25.Taurine; 26 Glucose; 27. Maltose; 28. Glycine; 29. Glycerol; 30. Fumarate; 31. Histamine; 32. Tyrosine; 33. Phenylalanine; 34. Urindine; 35. UDP; 36. Guanosine; 37. Hypoxanthine; 38. Formate; 39. Riboflavin; 40. Xanthine; 41. NADP^+^; 42. AMP; 43. ATP; 44. Nicotinurate; 45. Inosine; 46. Adenosine.


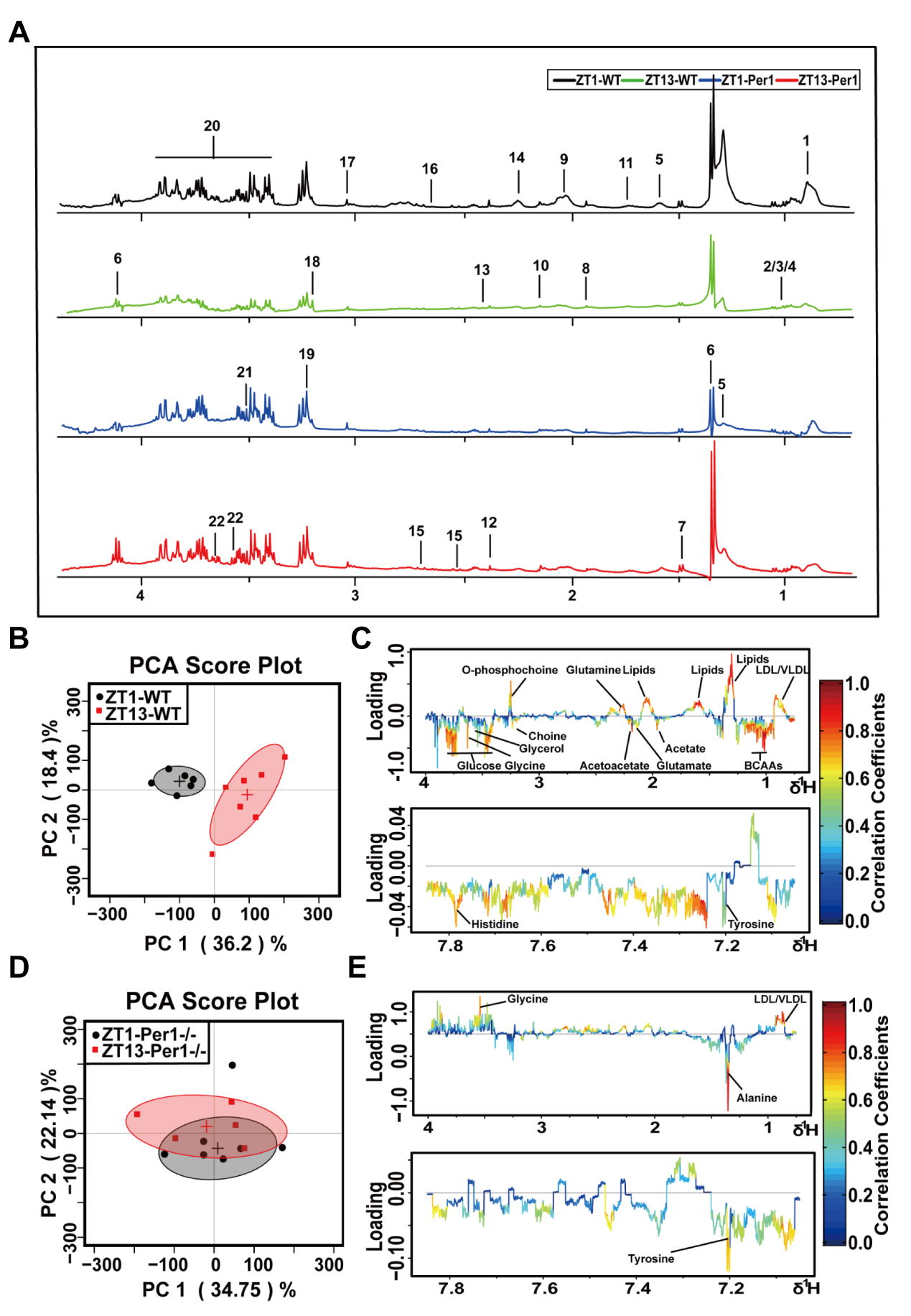


**Figure S2. OPLS-DA Analyses Based on ^1^H NMR Data from Serum Extracts of WT and *Per1^-/-^* mice.** A) Typical 500 MHz ^1^H NMR spectra of serum extracts with metabolites assigned. 1. LDL/VLDL; 2/3/4. BCAAs; 5. Lipids; 6. Lactate; 7. Alanine; 8. Acetate; 9. Glutamate; 10. Acetoacetate; 11. Lysine; 12. Pyruvate; 13. Succinate; 14. Glutamine; 15. Citrate; 16. Methionine; 17. Creatine; 18. Choline; 19. O-phosphocholine; 20. Glucose; 21. Glycine; 22. Glycerol; 23. Tyrosine; 24. Tropate; 25. Histidine. B,C) PCA scores plot (B) and color-coded loading plots (C) of OSC-PLS-DA for serum extracts obtained from WT mice (n=6-7/time point, 8 weeks old). D,E) PCA scores plot (D) and color-coded loading plots (E) of OSC-PLS-DA for serum extracts obtained from *Per1^-/-^* mice (n=5-6/time point, 8 weeks old). Metabolites that contributed to group separation were then visualized and color-coded according to the absolute correlation coefficient of each variable with each group. Color coded according to the fold change in metabolites, red indicated a significant change. Normalized values were shown in Table S2. Throughout, male mice for this experiment were maintained on standard chow.


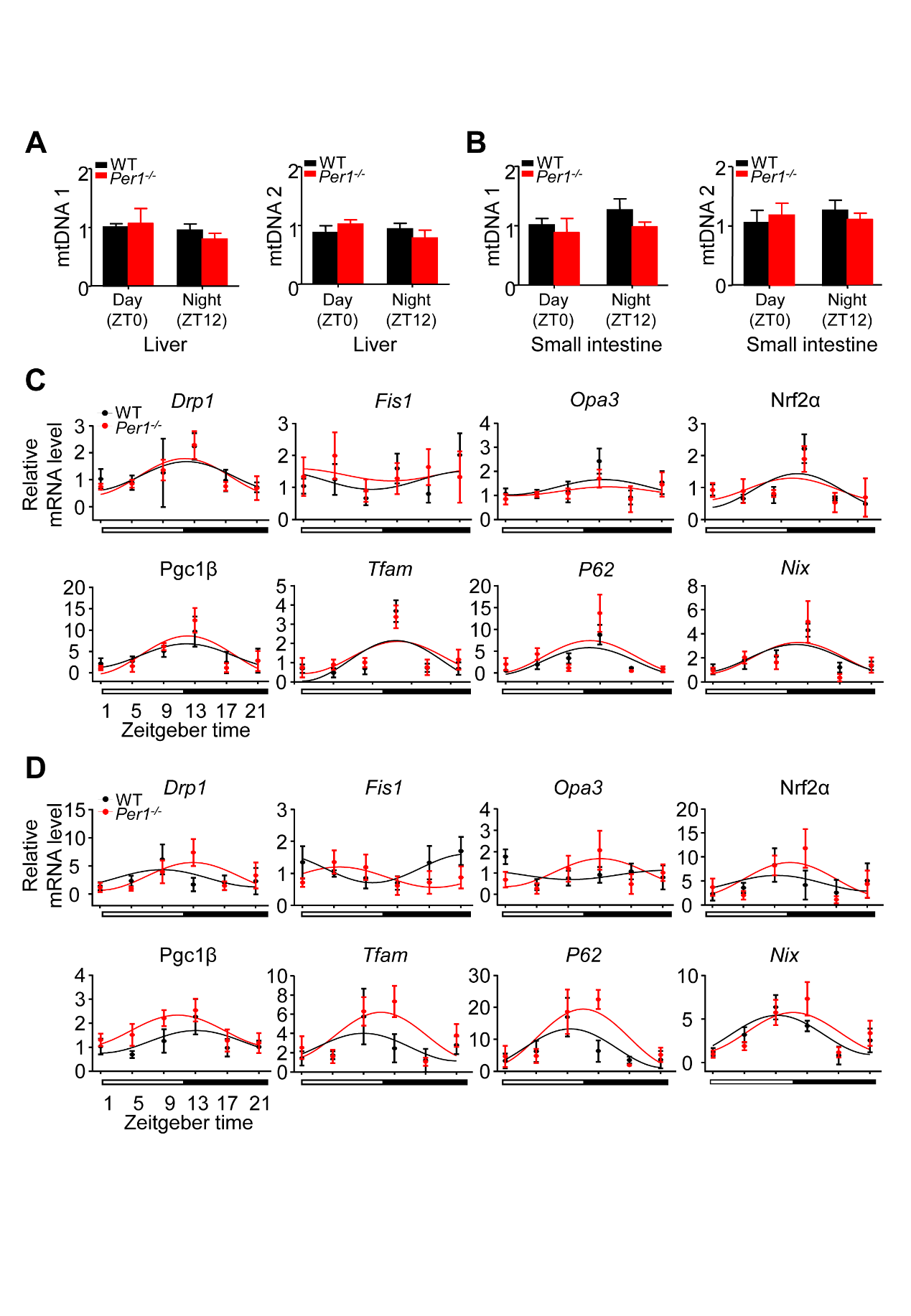


**Figure S3. The Mitochondrial DNA Copy Numbers and The Expression Profile of The Genes Involved in Mitochondrial Dynamics in WT and *Per1^-/-^* Mice.** (A,B) mitochondrial DNA copy numbers in the liver (A) and small intestine (B). Quantitative PCR using two sets of primers specific for mitochondrial DNA was performed and normalized for input using a control genomic locus in the liver and small intestine from male WT and *Per1^-/-^* mice (n=4/time point/genotype). No significant differences in mitochondrial DNA copy numbers were observed between WT and *Per1^-/-^* mice from day to night. C,D) Diurnal mRNA levels of the genes-related mitochondrial dynamics in livers (C) and small intestines (D) of WT (black) and *Per1^-/-^*(red) determined by quantitative real-time RT-PCR (n=4/time point/genotype). The white and black bar represents day and night, respectively. No obviously impaired expressions of the genes involved in mitochondrial dynamics in intestines were observed between WT and *Per1^-/-^* mice. Throughout, all data were expressed as the mean ± SEM and male mice for all experiments were maintained on standard chow. Analyses were performed using two-way ANOVA for A, B; and two-tailed Student's t-test for C, D in detailed Table S3.

**Table S1. Potential Marker Metabolites in Mouse Liver Identified by ^1^H NMR and Their Variations among Groups and the Associated *P*-values.**

| **NO.** | **Metabolites** | **Assignments** | | **ppm^a^** | **WT (Night) vs WT (Day)** | | ***Per1^-/-^*(Night) vs *Per1^-/-^*(Day)** | |
| --- | --- | --- | --- | --- | --- | --- | --- | --- |
|  |  |  |  |  | **Log_2_FC** | ***P* value** | **Log_2_FC** | ***P* value** |
| 1 | Cholate | 18CH3 | 0.74(s) | | 0.02 |  | -0.01 |  |
| 2 | Leucine | δCH3 , δCH3, γCH, αCH | 0.94(d), 0.96(d), 1.71(m), 3.74(m) | | -0.05 |  | -0.03 |  |
| 3 | Valine | γCH3, γCH3 | 0.98(d), 1.04(d), 2.26(m), 3.61(d) | | -0.06 |  | 0.05 |  |
| 4 | Isoleucine | δCH3 , γCH3 , αCH | 0.93(t), 1.0(d), 1.46(m) | | 0.05 |  | 0.04 |  |
| 5 | Isobutyrate | CH3 | 1.07(d) | | 0.04 |  | -0.04 |  |
| 6 | 3-Hydroxybutyrate | γCH3 | 1.18(d) | | 0.34 | ** | 0.18 |  |
| 7 | Lactate | CH3, CH | 1.33(d), 4.11(q) | | 0.86 | *** | 0.21 |  |
| 8 | Lipids | -(CH2)n-,-CH2CH2COOH | 1.3-1.38(m),1.58-1.7(m) | | 0.33 | * | 0.01 |  |
| 9 | Alanine | βCH3, αCH | 1.48(d), 3.78(q) | | 0.55 | ** | -0.02 |  |
| 10 | Lysine | δCH2, βCH2, ε-CH2 | 1.72(m),1.90(m),3.02(t) | | 0.16 |  | 0 |  |
| 11 | Acetate | CH3 | 1.92(s) | | -0.13 |  | 0.08 |  |
| 12 | N-Acetylcysteine | CH3 | 2.02(s) | | -0.29 | ** | -0.14 |  |
| 13 | Glutamate | βCH2,βCH2,γCH2, αCH | 2.14(m),2.36(m),2.50(m), 3.77(t) | | -0.12 |  | 0.13 |  |
| 14 | Succinate | CH2 | 2.4(s) | | 0.79 | ** | 0.13 |  |
| 15 | Glutamine | βCH2, γCH2, αCH | 2.46(m), 3.77(t) | | -0.23 |  | 0.06 |  |
| 16 | Sarcosine | CH3 | 2.74(s) | | 0.12 |  | 0.04 |  |
| 17 | Glutathione | S-CH2, N-CH, N-CH2,CH2 | 2.14(m), 2.55(m), 2.95(m) | | -0.82 | *** | -0.58 | * |
| 18 | Creatine | CH3, CH2 | 3.04(s),3.93(s) | | 0.21 |  | 0.02 |  |
| 19 | O-Acetylcarnitine | N-CH3 | 3.2(s) | | 0.09 |  | -0.04 |  |
| 20 | Choline | CH3 | 3.12(s) | | 0.34 |  | -0.12 |  |
| 21 | Carnitine | CH3 | 3.22(s) | | 0 |  | -0.08 |  |
| 22 | Myo-Inositol | CH | 3.27(t), 3.54(dd), 3.63(t), 4.07(t) | | -0.3 | * | -0.03 |  |
| 23 | Methanol | CH3 | 3.36(s) | | -0.46 |  | -0.55 |  |
| 24 | Glucose | 2H, 3H, 4H, 5H, 6H, 6’H | 3.4-3.95 (m), 5.24(d) | | 0.31 | * | 0.07 |  |
| 25 | Glycine | CH2 | 3.56(s) | | -0.99 |  | 0.16 |  |
| 26 | Taurine | NH2-CH2, SO3-CH2 | 3.28(t), 3.42(t) | | -0.05 |  | -0.42 | * |
| 27 | Maltose | CH, CH2 | 5.40 (m), 5.23 (d), 3.56-3.92 (m) | | -0.79 | ** | -0.32 |  |
| 28 | NAD | NH=CH-N | 9.35(s), 9.14(d), 8.82(d), 8.43(s), 8.17(m) | | -0.12 |  | 0.07 |  |
| 29 | Inosine | O-CH-N, N-CH=N,N-CH=N | 6.10 (d), 8.23 (s), 8.34 (s) | | 0.03 |  | 0.06 |  |
| 30 | Fumarate | CH=CH | 6.53(s) | | -0.23 |  | -0.15 |  |
| 31 | Histidine | 4'-CH, 2'-CH | 7.14 (s), 8.03 (s) | | -0.24 | ** | 0.01 |  |
| 32 | Tyrosine | H3/H5, C5H/C6H | 6.91(d),7.20(d) | | -0.14 |  | 0.02 |  |
| 33 | Phenylalanine | CH=CH | 7.38(m) | | -0.06 |  | -0.17 |  |
| 34 | UDP | CH, CH, CH | 5.79(d), 5.98(d), 7.96(d) | | -0.02 |  | -0.26 |  |
| 35 | Guanosine | O-CH-N, N-CH-N | 5.90 (d), 8.01 (s) | | -0.23 |  | -0.06 |  |
| 36 | Hypoxanthine | NH=CH-N, N=CH-NH | 8.18(s),8.20(s) | | 0.07 |  | -0.1 |  |
| 37 | Formate | CH | 8.44(s) | | -0.15 |  | -0.14 |  |
| 38 | ATP | N=CH-N, N=CH-N | 8.23(s),8.61(s) | | 0.53 | * | 0.04 |  |
| 39 | AMP | N=CH-N, N=CH-N | 8.23(s),8.55(s) | | -0.1 |  | -0.08 |  |
| 40 | Nicotinurate | 5’-CH, 6’-CH, 2’-CH | 7.6(d), 8.71(d), 8.94(d) | | 0.01 |  | 0 |  |
| 41 | NADP | NH=CH-N | 9.3(s), 9.09(d), 8.82(d), 8.13(s | | -0.11 |  | 0.07 |  |

^a^ Multiplicity: singlet (s), doublet (d), triplet (t), quartets (q), multiplets (m).

^b^ p-values corrected by BH (Benjamini Hochberg) methods were calculated based on a parametric Student’s t-test or a nonparametric Mann-Whitney test (dependent on the conformity to normal distribution). **p* < 0.05, ***p* < 0.01, ****p* < 0.001.

**Table S2. Potential Marker Metabolites in Mouse Serum Identified by ^1^H NMR and Their Variations among Groups and the Associated *P*-values.**

| **NO.** | **Metabolites** | **Assignments** | **ppm^a^** | **WT (Night) vs WT (Day)** | | | ***Per1^-/-^*(Night) vs *Per1^-/-^*(Day)** | |
| --- | --- | --- | --- | --- | --- | --- | --- | --- |
|  |  |  |  | **Log_2_FC** | ***P* value^b^** | | **Log_2_FC** | ***P* value** |
| 1 | LDL/VLDL | CH_3_，CH_2_ | 0.95(m),1.32(m) | -0.48 | | * | -0.62 |  |
| 2 | Leucine | δCH_3_ , δCH_3_, γCH, αCH | 0.94(d), 0.96(d), 1.71(m), 3.73(m) | 1.22 | | * | 0.36 |  |
| 3 | Isoleucine | δCH_3_ , γCH_3_ , αCH | 0.92(t), 1.03(d), 1.46(m) | 2.37 | | ** | -0.09 |  |
| 4 | Valine | γCH_3_, γCH_3_ | 0.99(d), 1.04(d),2.26(m) | 0.89 | | * | -0.24 |  |
| 5 | Lipids | -(CH_2_)n-,-CH_2_CH_2_COOH | 1.3-1.33(m),1.58-1.7(m) | -2.03 | | *** | -0.97 |  |
| 6 | Lactate | CH_3_, CH | 1.35(d), 4.12(q) | -0.7 | |  | 0.73 |  |
| 7 | Alanine | βCH_3_, αCH | 1.50(d), 3.77(q) | -0.18 | |  | 0.12 |  |
| 8 | Acetate | CH_3_ | 1.92(s) | 0.48 | |  | -0.39 |  |
| 9 | Glutamate | βCH_2_,βCH_2_,γCH_2_, αCH | 2.05(m),2.34(m),3.77(t) | -1.12 | | ** | -0.61 |  |
| 10 | Acetoacetate | CH_2_ | 2.29(s) | -0.97 | | * | -0.9 |  |
| 11 | Lysine | δCH_2_, βCH_2_, ε-CH_2_ | 1.72(m), 1.91(m), 3.00(t), 3.76(t) | 0.23 | |  | -0.26 |  |
| 12 | Pyruvate | CH_3_ | 2.39(s) | -0.34 | |  | -0.22 |  |
| 13 | Succinate | CH_2_ | 2.42(s) | 0.82 | |  | 0.18 |  |
| 14 | Glutamine | βCH_2_, γCH_2_, αCH | 2.16(m),2.45(m),3.77(t) | 0.74 | | * | 0.07 |  |
| 15 | Citrate | CH_2_ | 2.56(d),2.70(d) | -0.05 | |  | -0.74 |  |
| 16 | Methionine | CH_3_，CH_2_ | 2.14(s),2.63(t) | 0.49 | |  | -0.08 |  |
| 17 | Creatine | CH_3_, CH_2_ | 3.06(s),3.95(s) | 0.26 | |  | -0.41 |  |
| 18 | Choline | CH_3_ | 3.23(s) | 0.99 | |  | 0.38 |  |
| 19 | SG3P**^c^** | N(CH_3_)3 | 3.25(s) | -0.36 | |  | 0.37 |  |
| 20 | Glucose | 2H, 3H, 4H, 5H, 6H, 6’H | 3.35-3.98 (m), 4.65(d),5.24(d) | 0.6 | | * | 0.27 |  |
| 21 | Glycine | CH_2_ | 3.52(s) | 0.75 | |  | 0.13 |  |
| 22 | Glycerol | CH_2_, CH_2_, CH | 3.56(m),3.66(m),3.78(tt) | -0.46 | | * | -0.35 |  |
| 23 | Tyrosine | H_3_/H_5_, C_5_H/C_6_H | 6.93(d),7.24(d) | 0.29 | |  | -0.29 |  |
| 24 | Tropate | CH | 7.45(m) | 0.2 | |  | 0 |  |
| 25 | Histidine | 4'-CH, 2'-CH | 7.20 (s), 7.78 (s) | 0.78 | |  | -0.05 |  |

^a^ Multiplicity: singlet (s), doublet (d), triplet (t), quartets (q), multiplets (m).

^b^ p-values corrected by BH (Benjamini Hochberg) methods were calculated based on a parametric Student’s t-test or a nonparametric Mann-Whitney test (dependent on the conformity to normal distribution). **p* < 0.05, ***p* < 0.01, ****p* < 0.001.

**^c^** SG3P, sn-glycero-3-phosphocholine.

**Table S3. Rhythm Variations of WT and *Per1*^-/-^ Mice in the Liver and Small Intestine Related to Mitochondrial Dynamics.**

| **Gene** | **Mesor** | **Amplitude** | **Acrophase CT(h)** |
| --- | --- | --- | --- |
| Liver |  |  |  |
| WT |  |  |  |
| *Drp1* | 1.13±0.16 | 0.54±0.23 | 12.04±1.49 |
| *Fis1* | 1.22±0.14 | 0.28±0.21 | 21.75±2.75 |
| *Opa3* | 1.34±0.14 | 0.32±0.21 | 13.53±2.29 |
| *Nrf2α* | 0.9±0.12 | 0.53±0.18 | 12.15±1.18 |
| *Pgc1β* | 3.99±0.63 | 2.8±0.93 | 11.96±1.18 |
| *Tfam* | 1.09±0.23 | 1.06±0.34 | 12.99±1.11 |
| *P62* | 2.68±0.5 | 3.15±0.73 | 11.62±0.84 |
| *Nix* | 1.94±0.21 | 1.16±0.31 | 11.46±0.96 |
| *Per1* KO |  |  |  |
| *Drp1* | 1.1±0.12 | 0.68±0.17 | 11.81±0.92 |
| *Fis1* | 1.4±0.15 | 0.19±0.22 | 0.23±4.17** |
| *Opa3* | 1.17±0.11 | 0.18±0.16 | 13.99±3.29 |
| *Nrf2α* | 0.94±0.13 | 0.35±0.19 | 11.43±1.99 |
| *Pgc1β* | 4.13±0.8 | 4.49±1.17 | 12.08±0.96 |
| *Tfam* | 1.27±0.21 | 0.83±0.31 | 13.3±1.3 |
| *P62* | 3.69±1.08 | 3.71±1.56 | 11.73±1.57 |
| *Nix* | 1.96±0.42 | 1.33±0.59 | 11.81±1.68 |
| Small intestine |  |  |  |
| WT |  |  |  |
| *Drp1* | 2.83±0.46 | 1.49±0.63 | 8.98±1.76 |
| *Fis1* | 1.16±0.08 | 0.45±0.11 | 21.86±0.99 |
| *Opa3* | 0.91±0.14 | 0.22±0.18 | 21.29±3.48 |
| *Nrf2α* | 4.53±0.7 | 1.63±0.96 | 9.32±2.41 |
| *Pgc1β* | 1.23±0.12 | 0.47±0.17 | 13.07±1.49 |
| *Tfam* | 2.59±0.44 | 1.43±0.62 | 9.02±1.64 |
| *P62* | 7.22±1.18 | 6.04±1.56 | 9.4±1.11 |
| *Nix* | 3.17±0.38 | 2.25±0.52 | 9.21±0.96 |
| *Per1* KO |  |  |  |
| *Drp1* | 3.17±0.53* | 2.46±0.73 | 12.96±1.18 |
| *Fis1* | 0.88±0.09 | 0.32±0.13 | 5.69±1.41** |
| *Opa3* | 1.02±0.2 | 0.65±0.27 | 13.11±1.68 |
| *Nrf2α* | 5.41±0.91 | 3.46±1.24 | 11.04±1.45 |
| *Pgc1β* | 1.68±0.1* | 0.66±0.15 | 10.74±0.88 |
| *Tfam* | 3.75±0.53 | 2.46±0.75 | 11.27±1.15 |
| *P62* | 10.43±1.46 | 9.03±1.95 | 11.04±0.96 |
| *Nix* | 3.43±0.54 | 2.31±0.77 | 11.24±1.26 |

Values are mean ± SEM. CT indicates circadian time. Shown are rhythm-adjusted mean (mesor), amplitude, and peak time of rhythm (acrophase). *P<0.05, **P<0.01, *Per1* KO mice versus WT mice by two-tailed Student's t-test.

**Table S4. Rhythm Variations of WT and *Per1*^-/-^ mice in the liver and small intestine related to circadian clock genes.**

| **Gene** | **Mesor** | **Amplitude** | **Acrophase CT(h)** |
| --- | --- | --- | --- |
| Liver |  |  |  |
| WT |  |  |  |
| *Per1* | 6.61±1.27 | 8.73±1.79 | 13.18±0.76 |
| *Per2* | 5.28±0.4 | 5.01±0.58 | 14.79±0.42 |
| *Clock* | 0.88±0.05 | 0.33±0.07 | 22.74±0.88 |
| *Bmall* | 0.66±0.04 | 0.59±0.06 | 22.74±0.42 |
| *Cry1* | 1.11±0.07 | 0.82±0.1 | 19.03±0.46 |
| *Cry2* | 2.46±0.19 | 1.4±0.27 | 12.15±0.73 |
| *Per1* KO |  |  |  |
| *Per1* | - | - | - |
| *Per2* | 5.3±0.58 | 5.68±0.83 | 14.68±0.54 |
| *Clock* | 0.91±0.06 | 0.37±0.09 | 0.31±0.92 |
| *Bmall* | 0.72±0.08 | 0.57±0.12 | 22.59±0.76 |
| *Cry1* | 0.94±0.09 | 0.79±0.14 | 18.34±0.61 |
| *Cry2* | 2.66±0.17 | 1.19±0.23 | 11.04±0.8 |
| Small intestine |  |  |  |
| WT |  |  |  |
| *Per1* | 6.4±1.19 | 6.69±1.7 | 12.84±0.96 |
| *Per2* | 9.04±1.46 | 8.81±2.06 | 13.34±0.88 |
| *Clock* | 2.11±0.23 | 0.91±0.32 | 20.56±1.38 |
| *Bmall* | 2.07±0.39 | 2.09±0.54 | 22.01±1.03 |
| *Cry1* | 2.93±0.4 | 1.9±0.56 | 19.3±1.15 |
| *Cry2* | 4.7±0.55 | 1.93±0.8 | 14.79±1.53 |
| *Per1* KO |  |  |  |
| *Per1* | - | - | - |
| *Per2* | 11.55±6.01 | 13.64±8.5 | 13.76±2.37 |
| *Clock* | 1.89±0.29 | 1.23±0.42 | 12.31±1.22** |
| *Bmall* | 1.5±0.26 | 1.13±0.36 | 22.85±1.26 |
| *Cry1* | 2.85±0.62 | 1.71±0.87 | 16.13±1.95 |
| *Cry2* | 4.7±0.86 | 3.88±1.18 | 4.66±1.22** |

Values are mean ± SEM. CT indicates circadian time. Shown are rhythm-adjusted mean (mesor), amplitude, and peak time of rhythm (acrophase). *P<0.05, **P<0.01, Per1 KO mice versus WT mice by two-tailed Student's t-test.

**Table S5. The contribution of interface residues to the free energy of binding.**

|  | **Residue** | **Contribution (kcal/mol)** |
| --- | --- | --- |
|  | ARG 144 | -9.7933480 |
|  | PRO 134 | -3.4750856 |
|  | THR 128 | -3.3547672 |
| **GPX1** | ALA 129 | -3.2686508 |
|  | VAL 142 | -2.4810800 |
|  | LEU 130 | -2.2693096 |
|  | PRO 141 | -1.5862508 |
|  | THR 269 | -2.8810864 |
|  | MET 186 | -2.0488696 |
| **PER1** | TYR 310 | -1.9558716 |
|  | THR 308 | -1.6855688 |
|  | PHE 280 | -1.5416904 |

**Table S6. Key Resources used in the paper**

| **REAGENT or RESOURCE** | **SOURCE** | **IDENTIFIER** |  |  |
| --- | --- | --- | --- | --- |
| **Antibodies** | | |  |  |
| Glutathione Peroxidase 1 | Abcam | Cat#ab108427 |  |  |
| Glutathione Peroxidase 1 (For IF) | Abcam | Cat#ab22604 |  |  |
| HA-Tag | Cell Signaling | Cat# 3724 |  |  |
| PER1 | Abcam | Cat# ab3443 |  |  |
| β-actin | Bioworld | Cat# AP0060 |  |  |
| Rabbit IgG (Normal) | Beyotime | Cat#A7016 |  |  |
| HRP-labeled Goat Anti-Rabbit IgG | Beyotime | Cat#A0208 |  |  |
| Goat anti-Rabbit IgG (H+L) Cross-Adsorbed  Secondary Antibody, Alexa Fluor 568 | ThermoFisher | Cat#A-11011 |  |  |
| **Chemicals, Peptides, and Recombinant Proteins** | | |  |  |
| ATP | Sangon Biotech | Cat#A600020 |  |  |
| Saponin | Sigma | Cat# 47036 |  |  |
| ADP | Sigma | Cat# A2754 |  |  |
| NAD | Sigma | Cat#N3014 |  |  |
| Succinate | Sigma | Cat# W327700 |  |  |
| Xylenol orange | Aladin | Cat# X101229 |  |  |
| Ammonium ferrous sulfate | Sigma | Cat# F1543 |  |  |
| Sorbitol | Aladin | Cat# S104835 |  |  |
| MitoTracker™ Red CMXRos | ThermoFisher | Cat#M7512 |  |  |
| Recombinant Protein G-Sepharose™ 4B | ThermoFisher | Cat# 101241 |  |  |
| Recombinant Actin Beta | Cloud-Clone Crop | Cat# RPB340Mi01 |  |  |
| **Critical Commercial Assays** | | |  |  |
| Lipofectamine™ 2000 Transfection Reagent | ThermoFisher | Cat#11668019 |  |  |
| M-MLV Reverse Transcriptase | ThermoFisher | Cat# 28025013 |  |  |
| XbaI | NEB | Cat#R0145V |  |  |
| NotI | NEB | Cat#R0189V |  |  |
| BstXI | NEB | Cat#R0113V |  |  |
| EcoRI | NEB | Cat#R0101V |  |  |
| HindIII | NEB | Cat#R0104V |  |  |
| BamHI | NEB | Cat#R0136V |  |  |
| Q5® High-Fidelity DNA Polymerase | NEB | Cat#M0491S |  |  |
| T4 DNA Ligase | NEB | Cat#M0202T |  |  |
| SYBR Green Realtime PCR Master Mix | TOYOBO | Cat# QPK-101 |  |  |
| Glutathione Peroxidase (GSH-PX) assay kit | Nanjing Jiancheng Bioengineering Institute | Cat# A005 |  |  |
| Karrol Total RNA Isolation Kit | KarrotenScientific | Cat# K3102 |  |  |
| DNA Purification Kit | KarrotenScientific | Cat# K2201 |  |  |
| QIAamp DNA Mini Kit | QIAGEN | Cat#51304 |  |  |
| **Continued** | | |  |  |
| **REAGENT or RESOURCE** | **SOURCE** | **IDENTIFIER** |  |  |
| **Experimental Models: Cell Lines** | | |  |  |
| NIH-3T3 | ATCC | CRL-1658 |  |  |
| **Experimental Models: Organisms/Strains** | | |  |  |
| C57BL/6J *Per1^-/-^* | Gift of Dr. CC Lee | Reference [29] |  |  |
| C57BL/6 J | Nanjing Biomedical research Institute of Nanjing University | Cat#N000013 |  |  |
| **Oligonucleotides** | | |  |  |
| *Bmal1* oligos: | This paper | N/A |  |  |
| F: ACATAGGACACCTCGCAGAA | This paper | N/A |  |  |
| R: AACCATCGACTTCGTAGCGT | This paper | N/A |  |  |
| *Clock* oligos: | This paper | N/A |  |  |
| F: CGGCGAGAACTTGGCATT | This paper | N/A |  |  |
| R: AGGAGTTGGGCTGTGATCA | This paper | N/A |  |  |
| *Cry1* oligos: | This paper | N/A |  |  |
| F: TTCCCTGTTTCCTGACTCGT | This paper | N/A |  |  |
| R: GACAGCCACATCCAACTTCC | This paper | N/A |  |  |
| *Cry2* oligos: | This paper | N/A |  |  |
| F: TCGGCTCAACATTGAACGAA | This paper | N/A |  |  |
| R: GGGCCACTGGATAGTGCTCT | This paper | N/A |  |  |
| *Drp1* oligos: | This paper | N/A |  |  |
| F: AGAGCCCAGCCTACGGTGT | This paper | N/A |  |  |
| R: TCGGGGTGTTTTGTGTTGATA | This paper | N/A |  |  |
| R: CCCGGTCATTTGGTTTCTGC | This paper | N/A |  |  |
| *Fis1* oligos: | This paper | N/A |  |  |
| F: AAGTATGTGCGAGGGCTGTT | This paper | N/A |  |  |
| R: GGCAGAGAGCAGGTGAGG | This paper | N/A |  |  |
| *Actin* oligos: | This paper | N/A |  |  |
| F: GATCATTGCTCCTCCTGAGC | This paper | N/A |  |  |
| R: ACTCCTGCTTGCTGATCCAC | This paper | N/A |  |  |
| *Nampt* oligos: | This paper | N/A |  |  |
| F: ATTCCCGCCACAGTATCT | This paper | N/A |  |  |
| R: TCCCGATTGAAGTAAAGG | This paper | N/A |  |  |
| *Nix* oligos: | This paper | N/A |  |  |
| F: CGGGGGAACTCGACTTGTTG | This paper | N/A |  |  |
| R: CCATTGCTGCTGTTCATGGG | This paper | N/A |  |  |
| *Nrf2α* oligos: | This paper | N/A |  |  |
| F: CTCCCGCTACACCGACTAC | This paper | N/A |  |  |
| R: TCTGACCATTGTTTCCTGTTCTG | This paper | N/A |  |  |
| *Opa3* oligos: | This paper | N/A |  |  |
| F: GGCGAAGCTGTTCTACTTGG | This paper | N/A |  |  |
| **Continued** | | |  |  |
| **REAGENT or RESOURCE** | **SOURCE** | **IDENTIFIER** |  |  |
| R: CTGCTGCCTCCTCATTCAGT | This paper | N/A |  |  |
| *Per1* oligos: | This paper | N/A |  |  |
| F: TCCTCAACCGCTTCAGAGATC | This paper | N/A |  |  |
| R: CGGGAACGCTTTGCTTTAGA | This paper | N/A |  |  |
| *Per2* oligos: | This paper | N/A |  |  |
| F: GTGAAGCAGGTGAAGGCTAAT | This paper | N/A |  |  |
| R: AAGCTTGTAAGGGGTGGTGTAG | This paper | N/A |  |  |
| *Pgc1β* oligos: | This paper | N/A |  |  |
| F: CCATGCTGTTGATGTTCCAC | This paper | N/A |  |  |
| R: GACGACTGACAGCACTTGGA | This paper | N/A |  |  |
| *P62* oligos: | This paper | N/A |  |  |
| F: TGAAACATGGACACTTTGGCT | This paper | N/A |  |  |
| R: ACATTGGGATCTTCTGGTGGA | This paper | N/A |  |  |
| *Tfam* oligos: | This paper | N/A |  |  |
| F: CCACAGAACAGCTACCCAAATTT | This paper | N/A |  |  |
| R: TCCACAGGGCTGCAATTTTC | This paper | N/A |  |  |
| **Primer sequences used for cloning the Per1-EGFP expression vectors** | | |  |  |
| *Per1* fragment(BstXI/NotI) oligos: | This paper | N/A |  |  |
| F:CTGCAGAACCAGATTGGTGGAGGTTACTGAGTCGTCCAAT | This paper | N/A |  |  |
| R:ATAAGAATGCGGCCGCTGCTGGTGCTGTTTTCTTCTG | This paper | N/A |  |  |
| *Egfp* (NotI/XbaI) oligos: | This paper | N/A |  |  |
| F:ATAAGAATGCGGCCGCTACGTGTACGGTGGGAGGTCTA | This paper | N/A |  |  |
| R:CTAGTCTAGATTACTACGGTACCGTCGACTGCAGAAT | This paper | N/A |  |  |
| **Primer sequences used for cloning the HA-Per1(178-324 KO) expression vectors** | | |  |  |
| *Per1* fragment 1 (EcoRI/XbaI) oligos: | This paper | N/A |  |  |
| F:CCGGAATTCTTCACCTTCCCTGTTTCGTC | This paper | N/A |  |  |
| R:TGCTCTAGAAGCCTGAACCTGCTTGACAC | This paper | N/A |  |  |
| *Per1* fragment 2 (XbaI/HindIII) oligos: | This paper | N/A |  |  |
| F: TGCTCTAGAATTCGGGTCTCAGATGGAGC | This paper | N/A |  |  |
| R:CCCAAGCTTCTAAGCATAATCTGGAACATCATATGG | This paper | N/A |  |  |
| **Primer sequences used for cloning the HA-Per1(1056-1177 KO) expression vectors** | | |  |  |
| *Per1* fragment 1 (BstXI) oligos: | This paper | N/A |  |  |
| F:CTGCAGAACCAGATTGGTGGAGGTTACTGAGTCGTCCAATCA | This paper | N/A |  |  |
| R:GTCCACACACGCCGTCACTCGTGGGATCCT GAACCAGA | This paper | N/A |  |  |
| **Continued** | | |  |  |
| **REAGENT or RESOURCE** | **SOURCE** | **IDENTIFIER** |  |  |
| *Per1* fragment 2 (HindIII) oligos: | This paper | N/A |  |  |
| F:TCTGGTTCAGGATCCCACGAGTGACGGCGTGTGTGGAC | This paper | N/A |  |  |
| R:CCCAAGCTTCTAAGCATAATCTGGAACATCATATGG | This paper | N/A |  |  |
| **Primer sequences used for cloning the HA-Gpx1(full length: BamHI/XbaI) expression vectors** | | |  |  |
| F: CGCGGATCCATGTGTGCTGCTCGGCTC | This paper | N/A |  |  |
| R:CTAGTCTAGATTAAGCGTAGTCTGGGACGTCGTATGGGTAGGAGTTGCCAGACTGCTG | This paper | N/A |  |  |
| **Primer sequences used for cloning the HA-Gpx1(1-136: BamHI/XbaI) expression vectors** | | |  |  |
| F: CGCGGATCCATGTGTGCTGCTCGGCTC | This paper | N/A |  |  |
| R:CTAGTCTAGATTAAGCGTAGTCTGGGACGTCGTATGGGTAGGAGTTGCCAGACTGCTG | This paper | N/A |  |  |
| **Primer sequences used for cloning the HA-Gpx1(130-201: BamHI/XbaI) expression vectors** | | |  |  |
| F:CGCGGATCCATGGCCTTGCCAACACCCAGTGAC | This paper | N/A |  |  |
| R:CTAGTCTAGATTAAGCGTAGTCTGGGACGTCGTATGGGTAGGAGTTGCCAGACTGCTG | This paper | N/A |  |  |
| **Primer sequences** **used for recombinant fragments of the PER1 protein expression vectors** | | |  |  |
| **His-*Per1*fragment 208-414 (SalI /NotI)** | This paper | N/A |  |  |
| F:ACGCGTCGACATGCATCATCATCATCATCATATCACGTCTGAGTACACACTTCA | This paper | N/A |  |  |
| R:AAGGAAAAAAGCGGCCGCCTACTGCAGAATCTTCTTGTGGA | This paper | N/A |  |  |
| **His-*Per1* fragment 975-1290 (SalI /NotI)** | This paper | N/A |  |  |
| F:ACGCGTCGACATGCATCATCATCATCATCATTGCAGCTCTCCACTCCAGC | This paper | N/A |  |  |
| R:AAGGAAAAAAGCGGCCGCCTACTAGCTGGTGCAGTTTCCTG | This paper | N/A |  |  |
| **Mitochondrial DNA-specific primers** | | |  |  |
| mtDNA1 oligos: | This paper | Reference [37] |  |  |
| F: ACCATTTGCAGACGCCATAA | This paper |  |  |  |
| R: TGAAATTGTTTGGGCTACGG | This paper |  |  |  |
| mtDNA2 oligos: | This paper | Reference [37] |  |  |
| F: GCCCCAGATATAGCATTCCC | This paper |  |  |  |
| R: GTTCATCCTGTTCCTGCTCC | This paper |  |  |  |
| Actin DNA oligos: | This paper | Reference [37] |  |  |
| F: TGTTCCCTTCCACAGGGTGT | This paper |  |  |  |
| R: TCCCAGTTGGTAACAATGCCA | This paper |  |  |  |
| **Recombinant DNA** | | |  |  |
| **Continued** | | |  |  |
| **REAGENT or RESOURCE** | **SOURCE** | **IDENTIFIER** |  |  |
| pCMV-Sport2 Per1 Plasmid | Gift of Dr. CC Lee | Reference [14] |  |  |
| pCMV-Sport2 HA-Per1 Plasmid | Gift of Wang T | Reference [38] |  |  |
| pCMV-Sport2 Per1-GFP Plasmid | This paper | N/A |  |  |
| pCMV-Sport2 HA-Per1(178-324 KO) Plasmid | This paper | N/A |  |  |
| pCMV-Sport2 HA-Per1(1056-1177 KO) Plasmid | This paper | N/A |  |  |
| pcDNA3.1^+^ HA-Gpx1(full length) Plasmid | This paper | N/A |  |  |
| pcDNA3.1^+^ HA-Gpx1(1-136) Plasmid | This paper | N/A |  |  |
| pcDNA3.1^+^ HA-Gpx1(130-201) Plasmid | This paper | N/A |  |  |
| **Software and Algorithms** |  |  |  |  |
| GraphPad Prism 5 | GraphPad Software | http://www.graphpad.com/scientificsoftware/prism/ |  |  |
| Origin 7.0 | OriginLab | https://www.originlab.com/ |  |  |
| Image J | NIH | https://imagej.nih.gov/ij/download.html |  |  |
| Bruker Topspin 3.0 | Bruker | https://www.bruker.com/cn/service/support-upgrades/software-downloads/nmr.html |  |  |
| MestReNova 8.0.1 | Mestrelab esearch SL | http://mestrelab.com/download/mnova/ |  |  |
| Chenomx NMR Suite 7.5 | Chenomx Software | http://www.chenomx.com/software/ |  |  |
| R 3.3.3 | R Core Team | (http://cran.r-project.org/) |  |  |
